# To cleave or not to cleave: a systemic evaluation of DSS versus DSSO for cross-linking mass spectrometry analysis

**DOI:** 10.1038/s44320-026-00222-9

**Published:** 2026-06-09

**Authors:** Yong Cao, Peng-Zhi Mao, Qing-Cui Wu, Zhen-Lin Chen, Jin-Cai Yang, Yue Zhao, Adalet Memetimin, Hao Chi, Xiang-Bing Qi, Si-Min He, Meng-Qiu Dong

**Affiliations:** 1 National Institute of Biological Sciences, Beijing, Beijing, China; 2 Key Laboratory of Intelligent Information Processing of Chinese Academy of Sciences (CAS), Institute of Computing Technology, CAS, Beijing, China; 3 University of Chinese Academy of Sciences, Beijing, China; 4 Tsinghua Institute of Multidisciplinary Biomedical Research, Tsinghua University, Beijing, China

**Keywords:** Proteomics

## Abstract

Cross-linking mass spectrometry is a powerful method for structural analysis, but choosing between cleavable and non-cleavable cross-linkers remains challenging. We rigorously compared non-cleavable DSS with cleavable DSSO and found that DSS consistently yields more cross-link identifications from isolated protein complexes to bacterial lysates. The advantage of DSS diminishes as sample complexity increases. At the highest complexity tested—human cell lysate—the trend reverses, with DSSO outperforming DSS. The superior performance of DSS in less complex samples is likely explained by its longer and more flexible spacer arm, which interrogates a spatial volume >40% larger than that of DSSO. For both cross-linkers, the number of identified cross-links decreases as the search space expands, but more steeply for DSS. This sharper decline arises from DSS cross-links producing slightly lower fragment ion coverage, not from the absence of signature ions that could reduce search space. Fragment ion coverage is key to interactome mapping: when coverage reaches 85% or above, identification sensitivity hardly decreases as the search space expands, regardless of the cross-linker used. In summary, we recommend DSS for samples no more complex than bacterial lysates. For interactome mapping of mammalian cells, although DSSO outperforms DSS, neither achieves deep interactome coverage.

## Synopsis

This study compares non-cleavable DSS and cleavable DSSO across a range of sample complexities, showing that DSS yields more cross-link identifications in simpler samples, whereas DSSO performs better in highly complex mammalian whole-cell lysates.

- DSS probes a greater spatial volume and identifies more cross-links than DSSO in purified protein complexes, yeast ribosome, and bacterial lysate samples.
- Search space expansion reduces identification sensitivity more for DSS than for DSSO, due to slightly lower fragment ion coverage of DSS cross-links, not the absence of doublet ions.
- High fragment ion coverage of 85% or above maintains identification sensitivity regardless of search space size or cross-linker type.
- DSS is recommended over DSSO for samples no more complex than bacterial lysates.

## Abstract (web summary)

This study compares non-cleavable DSS and cleavable DSSO across a range of sample complexities, showing that DSS yields more cross-link identifications in simpler samples, whereas DSSO performs better in highly complex mammalian whole-cell lysates.

## Graphical abstract

**Figure:**
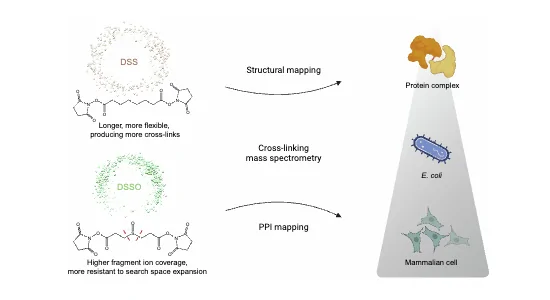

**Figure:**
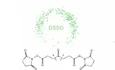

## Introduction

Chemical cross-linking of proteins coupled with mass spectrometry analysis (abbreviated as cross-linking mass spectrometry, XLMS, CXMS, or CLMS) is a cornerstone technique in structural proteomics. XLMS is developed to identify peptide pairs or residue pairs that are joined covalently through a chemical cross-linker, which is possible only when they are held in proximity by the rest of the protein or proteins to which they belong. As such, XLMS uncovers distance information between amino acid residues, which can be used for structural modeling of proteins or protein complexes (Gotze et al, 2019; Jin et al, 2022; Wheat et al, 2021; Wu et al, 2016), and even for in situ structure-function analysis (O’Reilly et al, 2020; Ryl et al, 2020). Applications of XLMS have expanded from analyzing relatively small complexes to megadalton complexes; from understanding static conformations to conformational dynamics (Liu et al, 2017b; Wang et al, 2022a; Zhao et al, 2024) and protein unfolding (Wang et al, 2022b). Presently, multiple laboratories are exploring XLMS for large-scale interactome mapping (Bogdanow et al, 2023; Chen et al, 2023; Zhu et al, 2024), which could bridge molecular details with systems-level biology.

**Figure 1:**
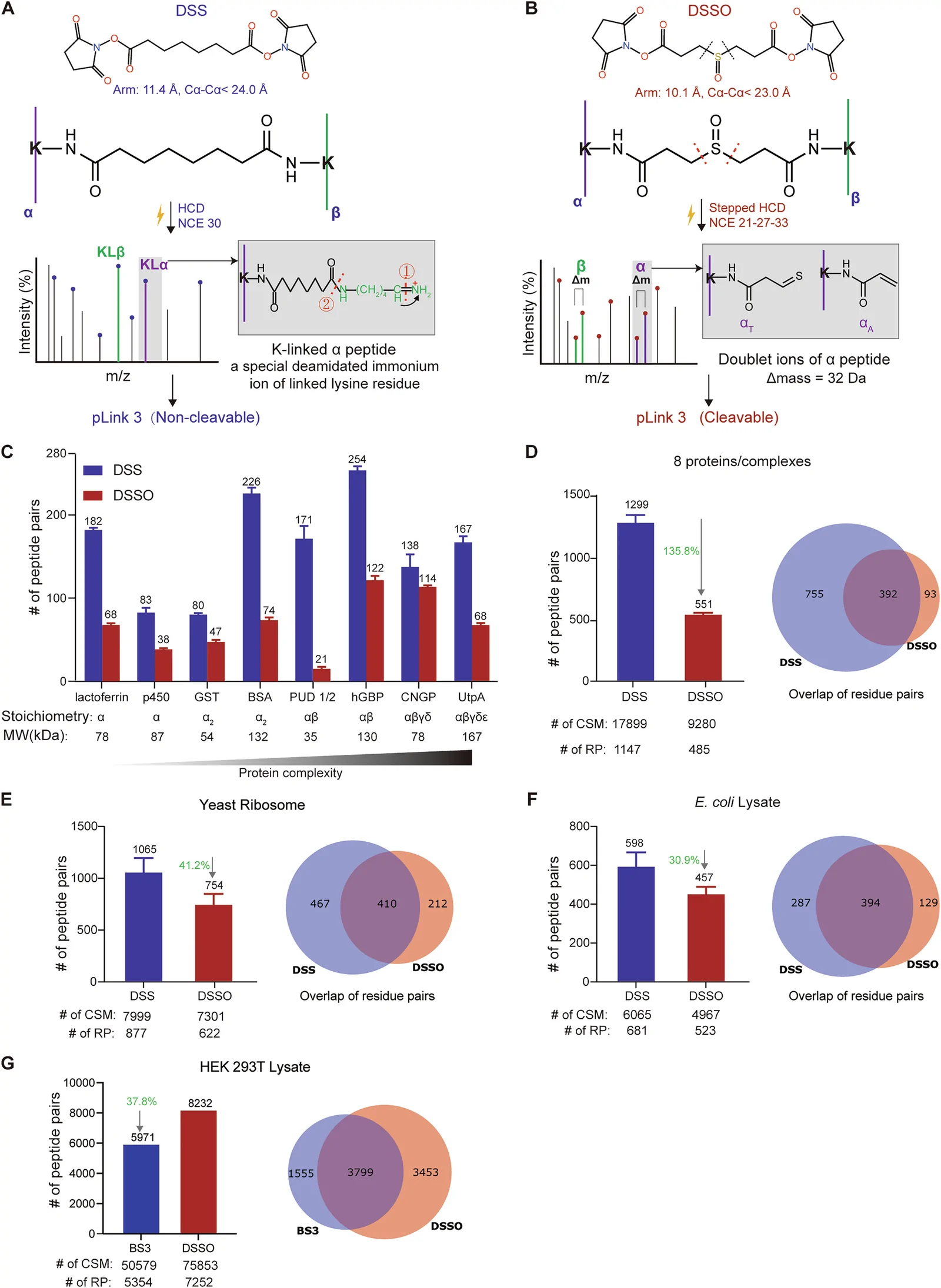
Performance evaluation of the identification workflows for DSS and DSSO cross-links. (**A**, **B**) Illustration of the optimized workflow for DSS (**A**) or DSSO (**B**) cross-links. (**C**–**F**) Comparison of DSS and DSSO cross-links identified from samples of low-, medium-, and high-complexity, represented by eight isolated proteins or protein complexes (**C**, **D**), yeast ribosome (**E**), and *E. coli* lysate (**F**), respectively. Data are presented as mean ± SD (*n* = 4, two biological replicates, each of two technical repeats). (**G**) Comparison of BS3, a surrogate of DSS with higher solubility, and DSSO for interactome mapping using cultured human HEK293T cells. The cells were lysed, cross-linked with BS3 or DSSO, digested, and fractioned by strong cation exchange (SCX) chromatography. For each cross-linker, 36 SCX fractions were analyzed for identification of cross-linked peptide pairs. Source data are available online for this figure.

The success of XLMS relies heavily on the cross-linker used. A cross-linker may have multiple functional groups, but in its most basic form it consists of two protein-targeting chemical groups (sometimes referred to as “warheads”) separated by a spacer arm (two examples shown in Fig. 1). As warheads, N-hydroxysuccinimide (NHS) esters are widely favored for their selective reactivity towards primary amines under physiological conditions (Chen et al, 2025; Kao et al, 2011; Matzinger and Mechtler, 2021; Muller et al, 2010; Tan et al, 2016; Young et al, 2000). A spacer arm can be a plain carbon chain whose strong C-C bonds remain intact during collision-induced dissociation (CID), or one that contains weaker bonds to cause a cross-linker to break apart upon CID, thereby releasing the two cross-linked peptides in the gas phase (Ianni et al, 2024; Kao et al, 2011; Muller et al, 2010; Wheat et al, 2021).

Among the NHS ester-based homobifunctional cross-linkers, disuccinimidyl suberate (DSS) serves as a prototypical non-cleavable cross-linker (Fig. 1A), while disuccinimidyl sulfoxide (DSSO), with its gas-phase-cleavable C-S bonds, represents a popular choice for a cleavable cross-linker (Fig. 1B). The two symmetric C-S bonds in the spacer arm of DSSO are equally susceptible to cleavage by CID, resulting in a pair of ions with a characteristic mass difference (Δm) for each of the released peptides, that is, the doublet peptide ions. Based on this feature, specialized data acquisition methods (e.g., multi-stage fragmentation) and dedicated search algorithms are developed to identify DSSO-linked peptide pairs (Birklbauer et al, 2023; Clasen et al, 2024; Gotze et al, 2019; Liu et al, 2017a; Liu et al, 2015). DSSO and DSS, as well as the sulfonated form of DSS (Bis[sulfosuccinimidyl] suberate, BS3), are all benchmark reagents in their respective categories (Steigenberger et al, 2020).

Although these cross-linkers are widely used, selecting the appropriate one for an experiment can be challenging. The choice between non-cleavable (e.g., DSS) and cleavable (e.g., DSSO) cross-linkers effectively represents a choice between two distinct sets of XLMS workflows and must be evaluated based on experimental objectives, reagent costs, instrumental requirements, and data analysis capabilities. However, the non-cleavable workflows and the cleavable workflows are not evaluated sufficiently, partly because the latter ones have many variations (Muller et al, 2025; Petrotchenko et al, 2011; Wheat et al, 2021). Empirically, a cleavable workflow is usually more expensive due to proprietary reagents and may require advanced mass spectrometers capable of multi-stage fragmentation and specialized software (Liu et al, 2017a). From the literature, it appears that non-cleavable cross-linkers such as DSS are used more often for low- and medium-complexity samples, while cleavable cross-linkers such as DSSO are frequently used for high-complexity samples such as cell lysates (Gonzalez-Lozano et al, 2020; Gotze et al, 2019; Makepeace et al, 2020; Yugandhar et al, 2020). Whether this reflects fundamental differences between the two sets of workflows needs formal investigation.

To provide evidence-based guidelines for workflow selection, we systematically benchmarked the performances of a standard DSS workflow and a carefully optimized DSSO workflow involving higher-energy collisional dissociation with stepped collision energy (stepped HCD). Our results show that the DSS workflow consistently identified more cross-links than the DSSO workflow on purified proteins, small or large protein complexes, and an *E. coli* lysate. The superior performance of the DSS workflow can be attributed to the longer and more flexible spacer arm of DSS compared to DSSO. By simple calculation or by molecular dynamics simulation, the volume of space that can be reached by DSS for cross-linking is at least 40% greater than that by DSSO. However, the advantage of the DSS workflow decreases as sample complexity increases. We show that this trend cannot be explained by the absence of doublet ions or other signature ions in MS/MS spectra that could be used to narrow down candidate sequences for identification of DSS-linked peptide pairs, or DSS cross-links. Instead, the slightly lower median sequence coverage by fragment ions of DSS cross-links—compared to those of DSSO cross-links—is responsible for a greater sensitivity loss to search space explosion. This is consistent with the finding of a previous study (Kolbowski et al, 2022). The above results suggest that DSS produces more cross-links than DSSO, but DSS cross-links—especially inter-protein cross-links—are more difficult to identify if their MS/MS spectra are searched against a large database of thousands or tens of thousands of proteins.

## Results

### Optimization of a DSSO workflow

To ensure an unbiased and rigorous comparison between DSS and DSSO, both cross-linkers must be evaluated under optimized data acquisition and data analysis conditions. Only then can the performance difference be attributed to the intrinsic differences between the two cross-linkers.

We have long focused on method development around DSS and other non-cleavable cross-linkers (Jin et al, 2022; Jones et al, 2019; Tan et al, 2016; Yang et al, 2012). We are thus confident about our DSS workflow. The pLink software, which we developed, is consistently the top performer in the category of non-cleavable cross-linkers (Chen et al, 2019; Lu et al, 2015; Yang et al, 2012). In this study, we used pLink 3 (Manuscript in preparation; https://github.com/pFindStudio/pLink3), an updated version of pLink 2, capable of identifying both DSS and DSSO cross-links with false discovery rate (FDR) control at multiple levels, from peptide pairs to protein pairs.

To make sure that we have an equally optimized DSSO workflow, we systematically explored data acquisition and data analysis parameters.

A plethora of data acquisition strategies had been employed for DSSO cross-links before HCD—particularly stepped HCD at normalized collisional energy of 21%-27%-33% (NCE 21-27-33) (Stieger et al, 2019)—was shown to be the best. We also evaluated different NCE levels and their combinations in finer grades and arrived at the same conclusion that stepped HCD of NCE 21-27-33 is optimal on Orbitrap Fusion Lumos (Appendix Figs. S1A–F and S2A–D). Under this condition, the intensity of signature doublet ions of DSSO cross-links and the sequence coverage by fragment ions (i.e., fragment ion coverage) of both the α and the β-peptides are kept at the top level.

For analysis of DSSO data, we implemented a new, dedicated module within the framework of pLink 3. Tested on the benchmarked public datasets of DSSO and DSBU (Beveridge et al, 2020), the cleavable module of pLink 3 displayed an excellent FDR control on par with Scout, a new and powerful search engine for cleavable cross-linkers (Clasen et al, 2024). Concerning the sensitivity of cross-link identification, the cleavable module of pLink 3 outperformed Scout by identifying 53–368% more cross-link-spectrum matches (CSMs) or 7–54% more cross-linked peptide pairs (Appendix Fig. S3A,B).

With the above optimization, we proceeded to a direct comparison of DSS and DSSO.

### DSS cross-links outnumbered DSSO cross-links for samples less complex than mammalian whole-cell lysates

We first compared the DSS workflow (Fig. 1A) and the DSSO workflow (Fig. 1B) on purified, low-complexity samples (Fig. 1C), ranging from monomeric proteins (e.g., lactoferrin and p450) to small, multi-subunit complexes (e.g., CNGP and UtpA). All analyses were conducted with two biological replicates, each of two technical repeats. The results showed unequivocally that across the eight samples tested, the numbers of cross-linked peptide pairs identified by the DSS workflow markedly exceeded those by the DSSO workflow, ranging from 21% (CNGP) to 714% (PUD1/2) (Fig. 1C). In total, 1299 nonredundant cross-linked peptide pairs were identified using DSS, and 551 using DSSO (Fig. 1D). These cross-linked peptide pairs corresponded to 1147 and 485 residue pairs for DSS and DSSO, respectively, with an overlap of 392 (Fig. 1D). In other words, the DSS workflow covered 81% of the residue pairs identified using DSSO, whereas for the other way around this number was 34%.

Next, we compared the DSS and DSSO workflows on medium- to high-complexity samples. The yeast ribosome is a 4.2 MDa macromolecular assembly composed of nearly 80 proteins. From this sample, the DSS workflow and the DSSO workflow identified 1065 and 754 cross-linked peptide pairs, respectively, with the former outperforming the latter by 41.2% (Fig. 1E). To further increase sample complexity, we used a whole-cell lysate of *E. coli* cells containing thousands of proteins. Still, the DSS workflow maintained its lead by identifying 598 cross-linked peptide pairs, which is 31% more than that by the DSSO workflow (Fig. 1F).

This trendline predicts that on samples of extreme complexity, e.g., mammalian whole-cell lysates, DSS may lose its edge to DSSO. Attempts to test this idea were made difficult by the limited solubility of DSS, as it cannot reach a high enough concentration necessary for cross-linking lysates of mammalian cells, in which more than 10,000 proteins are present. We thus turned to BS3 as a proxy for DSS, whose cross-linking products are identical in chemical structure. Indeed, the use of BS3 or DSSO led to a total of 5971 or 8232 cross-linked peptide pairs being identified, respectively, from a lysate of cultured human embryonic kidney (HEK293T) cells (Fig. 1G).

The above side-by-side comparisons showed that DSS cross-links outnumbered DSSO cross-links. However, as sample complexity increased, the advantage of DSS progressively narrowed, from 135% on simple protein complexes to 41% on yeast ribosome, and then to 31% on *E. coli* lysate. In the HEK293T lysate, this trend was reversed, with DSSO identifying 38% more cross-linked peptide pairs (Fig. 1C–G).

### The longer and more flexible spacer arm of DSS may account for its superior performance over DSSO

**Figure 2:**
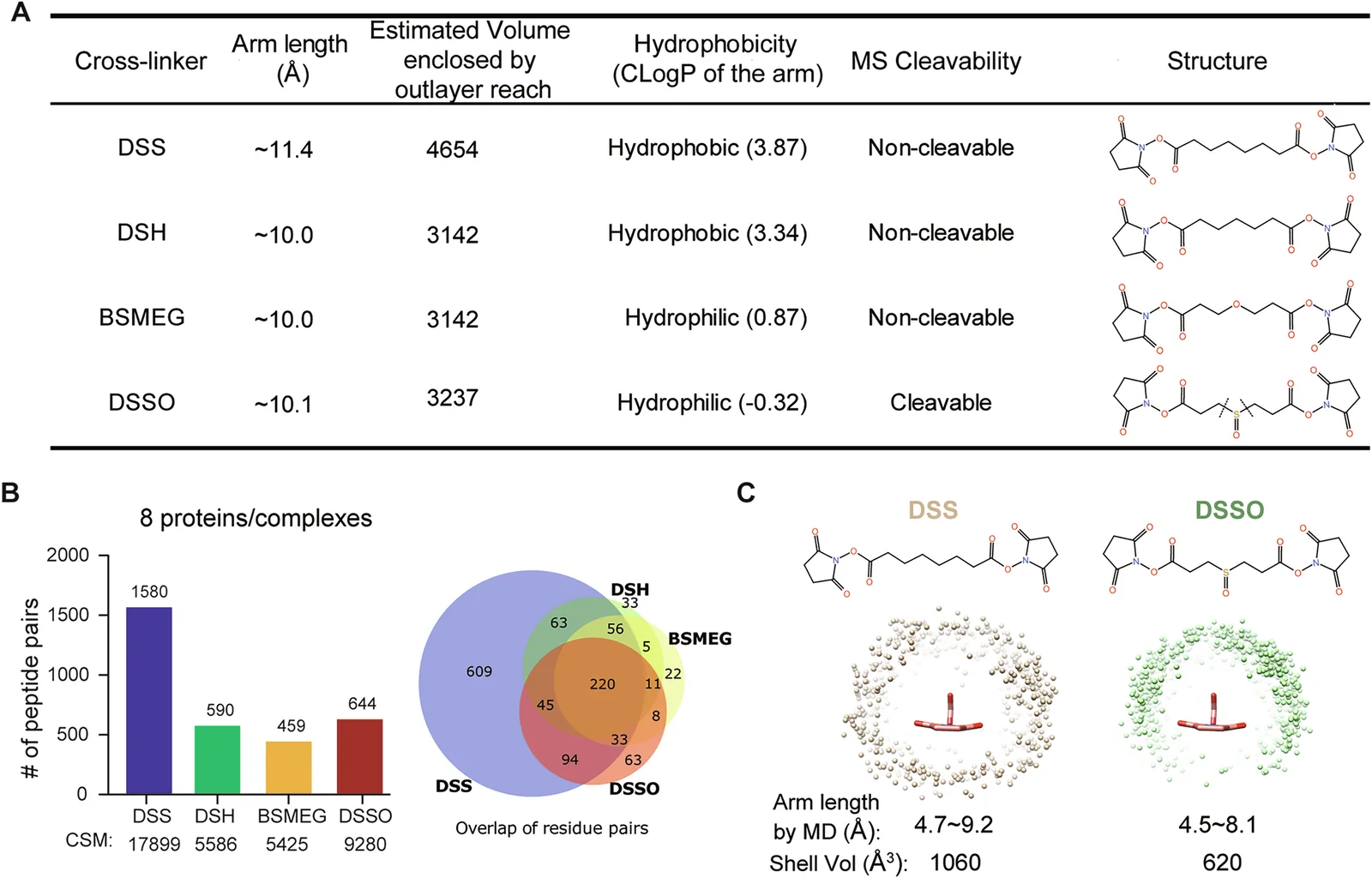
Analysis of the physicochemical properties of DSS, DSH, BSMEG, and DSSO and their effect. (**A**) Systematic comparison of the key physicochemical properties for the four cross-linkers used to deconvolve the effects of arm length, hydrophobicity, and cleavability. (**B**) The impact of these properties on cross-linking performance. The bar plot shows the total number of unique cross-linked residue pairs identified for each reagent. The Venn diagram illustrates the overlap between the identified sets, highlighting the superior capture efficiency of DSS. (**C**) Mechanistic insight into the role of spacer arm flexibility. Molecular dynamics simulation shows the accessible conformational space for a fixed DSS (yellow) and DSSO (green) cross-linker. The larger, more uniform sampling volume of DSS is quantified below, along with its effective arm length range, providing a structural basis for its enhanced performance. Source data are available online for this figure.

To find out what lies beneath the superior performance of DSS over DSSO, we examined the physicochemical properties of the two cross-linking reagents, including spacer arm length, hydrophobicity, and MS-cleavability. To delineate these factors, a controlled experiment was carried out with two additional non-cleavable cross-linkers: disuccinimidyl heptanoate (DSH) and bis(succinimidyl) mono(ethylene glycol) (BSMEG). As shown in Fig. 2A, DSH is almost an exact copy of DSS except that its spacer arm is shorter by one carbon atom. At 10.0 Å, the spacer arm of DSH and that of BSMEG are of a similar length to DSSO. BSMEG resembles DSSO in spacer arm length and hydrophobicity, but unlike DSSO, it is non-cleavable. In sum, by comparing DSS to DSH, DSH to BSMEG, and BSMEG to DSSO, we can isolate the effect of spacer arm length, hydrophobicity, and MS-cleavability, respectively (Fig. 2A).

We performed this comparative analysis on the same proteins or protein complexes as shown in Fig. 1C. From the eight samples, a total of 590, 459, and 655 cross-linked peptide pairs were identified using DSH, BSMEG, and DSSO, respectively, far less than 1580—the number of cross-links identified using DSS (Fig. 2B). Furthermore, we examined the number of mono-links, that is, DSS or DSSO modified but not cross-linked peptides, across four datasets (the eight purified proteins or complexes as a group, yeast ribosome, *E. coli*, and HEK293T lysates) and found that the numbers were similar between DSS and DSSO (Appendix Fig. S4A–H). This suggests the NHS ester groups in DSS and DSSO have comparable labeling efficiencies. We thus conclude that the longer spacer arm of DSS—not hydrophobicity or MS-cleavability—could account for the performance difference between DSS and DSSO.

Although the spacer arm of DSS (11.4 Å) is only 12.9% longer than that of DSSO (10.1 Å), this seemingly small difference is augmented in 3D space. Under a simple assumption that each cross-linker, with one end anchored and the other accessing freely a spherical space limited only by the length of its spacer arm, the volume accessible by DSS is 43.8% greater than that of DSSO, or 48.1% greater than DSH and BSMEG (Fig. 2A).

It did not escape our attention that the spacer arm of DSS, all sigma-bonded, is more flexible than that of DSSO, which has a S=O bond. Molecular dynamics simulation was carried out by anchoring one end of each cross-linker and modeling the accessible positions of the reactive NHS ester on the other end. Similar analyses have been performed previously (Gong et al, 2020; Zhou et al, 2025). The simulation result revealed that for both DSS and DSSO, the most readily accessible 3D space resembles a spherical shell, but there are differences (Fig. 2C; Appendix Fig. S5). For DSS, the shell is larger and more uniform. In contrast, for DSSO the shell is smaller with a noticeable gap in it. The estimated shell volumes of DSS and DSSO are 1060 and 620 cubic angstroms, respectively. This means that the longer and more flexible spacer arm allows DSS to access a 3D space 71.9% greater than what is accessible by DSSO. This difference (71.9%), based on computational modeling, is more pronounced than that (43.8%) by simple calculation, as described above.

SDS-PAGE analysis following DSS or DSSO cross-linking of homo-dimeric proteins corroborated the above results. As shown, although treatment with either cross-linker shifted BSA (Appendix Fig. S6A) and GST (Appendix Fig. S6B) towards higher molecular weight positions, including those of dimers, with a corresponding depletion of the monomeric band, this effect was markedly stronger with DSS than with DSSO.

Based on all the data above, we conclude that the longer and more flexible spacer arm grants DSS access to a substantially greater number of potential cross-linking sites, thereby producing more protein cross-links than DSSO under the same conditions.

### The lack of α- or β-peptide ions in MS2 does not explain the sample complexity-associated performance decline of DSS

Next, we investigated why the performance advantage of the non-cleavable DSS workflow narrows as sample complexity increases (Fig. 1). This trend suggests that cleavable reagents like DSSO may possess a latent advantage that only manifests when experimental or computational conditions become complex.

A prevailing perception holds that the key to the success of DSSO as a cross-linker is the production of signature doublet ions upon MS2 (Kao et al, 2011; Liu et al, 2015; Piersimoni et al, 2022; Yu and Huang, 2018). These doublet ions inform about the mass of the α- or β-peptide, or both, thereby enabling information-driven filtering of candidate peptides to reduce search space. This is particularly welcome in the analysis of high-complexity samples such as lysates of mammalian cells or tissues, of which the search space becomes extremely large (Jagtap et al, 2013; Parfentev et al, 2020). It is believed that under such circumstances, reducing the search space would increase identification sensitivity. However, a recent study challenged this view by showing that filtering of candidate peptides by way of doublet ions does not increase identification sensitivity because production of doublet ions is incomplete—up to 10% of the MS2 spectra of DSSO cross-links have none (Kolbowski et al, 2022).

Here, we investigated whether the lack of peptide ions is a disadvantage of DSS that takes a heavier toll on high-complex samples. If true, it should not happen to MS2 spectra with peptide ions spiked in computationally. DSS-linked peptide pairs sometimes generate a K-linked α- or β-peptide ion (KLα/β), or both, along with the deamidated species KLα/β-NH_3_ (Fig. 1A, inset) (Steigenberger et al, 2019b; Yang et al, 2012). KLα/β-NH_3_ ion can be found in about 67.6% of the HCD spectra of DSS cross-links that we have examined (Appendix Fig. S7A,B).

**Figure 3:**
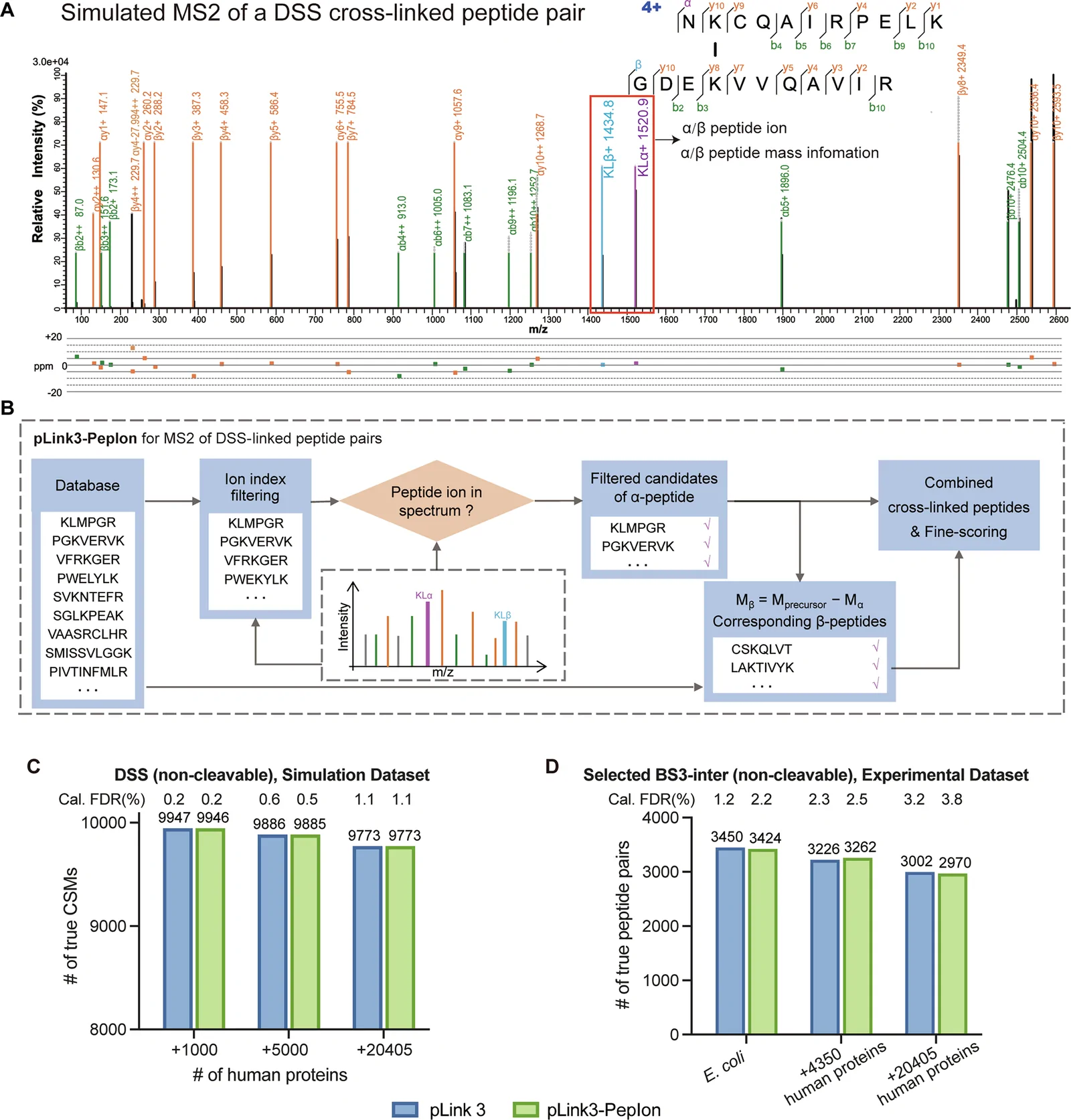
Adding signature α- and β-peptide ions to MS2 of DSS cross-links fails to overcome the identification sensitivity loss caused by search space expansion. (**A**) An example of simulated MS2 spectrum of a DSS-linked peptide pair, which is computationally generated to guarantee the presence of K-linked peptide ions that report the mass of the α- and β-peptides. (**B**) The search flow of pLink3-PepIon, which filters candidate α- and β-peptides based on K-linked peptide ions. (**C**, **D**) Performance comparison of the pLink3-PepIon search flow and the standard pLink 3 search flow, which does not filter candidate peptides based on K-linked peptide ions. (**C**) A simulated MS2 dataset of DSS cross-links and (**D**) a real-world dataset consisting of MS2 of *E. coli* inter-protein cross-links (PXD019120) were used. Source data are available online for this figure.

We therefore simulated a set of MS2 spectra of DSS cross-links with KLα and KLβ ions spiked into each spectrum (Fig. 3A). We also designed a pLink3-PepIon workflow, which filters candidate peptide sequences based on the KLα/β ions detected (Fig. 3B). These simulated MS2 spectra were searched against the target sequences along with 1000 to all (20,405) sequences of human proteins. As shown, the search results of pLink3-PepIon and pLink 3—which makes no use of the KLα/β ions—were essentially the same (Fig. 3C). We further curated a collection of experimental spectra containing KLα/β ions. Replacing the simulated MS2 spectra with these experimental spectra did not change the outcome (Fig. 3D).

Hence, the lack of peptide ions and the lack of filtering of candidate peptides based on these ions is not the reason why the performance advantage of DSS over DSSO weakens on high-complexity samples.

### Filtering candidate peptides based on signature doublet ions decreased the identification sensitivity of DSSO cross-links

**Figure 4:**
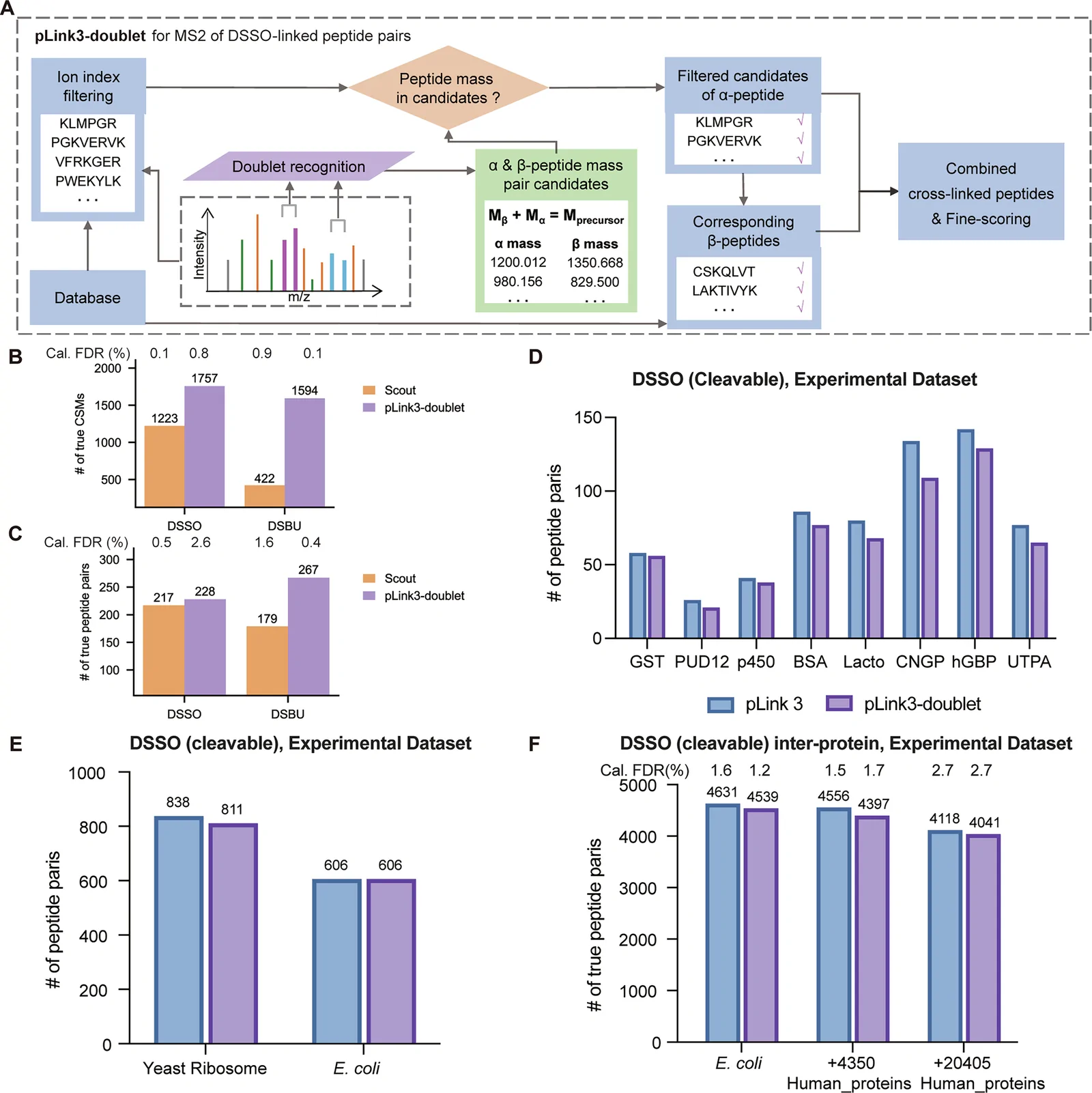
Not using doublet ions for filtering candidate α- and β-peptides of DSSO cross-links has no negative effect on cross-link identification. (**A**) Schematic representation of the doublet-based filtering strategy employed by the pLink3-doublet algorithm, in which signature doublet ions are used to calculate the α- and β-peptide masses prior to database searching. (**B**, **C**) Validation and benchmarking of the pLink3-doublet algorithm against the Scout search engine using data of synthetic peptides (PXD014337) cross-linked with DSSO or DSBU. Identified CSMs (**B**) or peptide pairs (**C**) are displayed at the indicated FDR levels. (**D**–**F**) Performance comparison of pLink 3, which does not filter candidate peptides based on signature doublet ions, versus pLink3-doublet. Test datasets of DSSO cross-links include (**D**) isolated proteins or protein complexes, (**E**) yeast ribosome and *E. coli* lysate, and (**F**) selected *E. coli* inter-protein cross-links (PXD019120). The search space of (**F**) was expanded by adding human proteins. Source data are available online for this figure.

The DSSO workflow of pLink 3, which we have used up till this point, does not use the information carried by doublet ions to filter candidate peptides. To examine how this might affect search results, we developed pLink3-doublet, which does take advantage of the well-known 32 Da mass difference of the signature doublet ions for the purpose of search space reduction (Fig. 4A). Based on the detected doublet ions, pLink3-doublet calculates the molecular weight of candidate α- and β-peptides and uses them to query the protein sequence database to retrieve candidate peptide sequences for fine scoring. If no doublet ions are detected in a MS2 spectrum, the spectrum is treated as a non-cross-link and thus discarded.

We evaluated pLink3-doublet against Scout on two synthetic peptide datasets (Beveridge et al, 2020) (Fig. 4B,C). On the DSSO dataset, pLink3-doublet outperformed Scout by 43.7% or 5.1% on the number of CSMs or identified peptide pairs, respectively (Fig. 4B). On the DSBU dataset, the advantage of pLink3-doublet over Scout was more pronounced (Fig. 4C). Of either dataset, 96.3% or more of the cross-linked peptide pairs identified by Scout were among those identified by pLink3-doublet (Appendix Fig. S8).

We then compared pLink3-doublet with pLink 3 to isolate the effect of candidate peptide filtering based on doublet ions. From simple proteins or protein complexes to yeast ribosomes and *E. coli* lysate, pLink3-doublet failed to increase the sensitivity of identification (Fig. 4D,E). Rather, slightly but consistently, it decreased the number of identified cross-links. This is in perfect agreement with the findings of an earlier study (Kolbowski et al, 2022) and can be explained by imperfection of experimental spectra—~10% of valid DSSO cross-link spectra lack doublet ions.

Lastly, we reasoned that the presumed benefits of doublet ions, namely, candidate peptide filtering and ensuing search space reduction, would be most apparent in the scenario of identifying inter-protein cross-links from a massively expanded search space. We thus analyzed a published, large-scale dataset of *E. coli* inter-protein cross-links (Lenz et al, 2021) against a database containing not only the *E. coli* proteins but also a varying number of human proteins (Fig. 4F). Even under the condition where all 20,405 human protein sequences were appended to the *E. coli* protein database, designed to maximally favor the doublet-based filtering strategy, fewer cross-links were identified using the filtering strategy than not (Fig. 4F).

In sum, this study joins an early one (Kolbowski et al, 2022) to refute the idea that using signature peptide ions to reduce search space can improve identification sensitivity. On the contrary, it slightly decreases identification sensitivity due to erroneous exclusion of authentic cross-link spectra.

### Slightly lower fragment ion coverage of DSS cross-links has a negative effect on identification sensitivity that manifests when the search space explodes

Since the presence or absence of signature peptide ions cannot explain why the performance advantage of DSS over DSSO diminishes on high-complexity samples, we looked for alternative reasons. It has been proposed that better peptide backbone fragmentation is the primary advantage of DSSO over BS3 (Kolbowski et al, 2022; Liu et al, 2015), but a quantitative cause–effect relationship has not been established.

**Figure 5:**
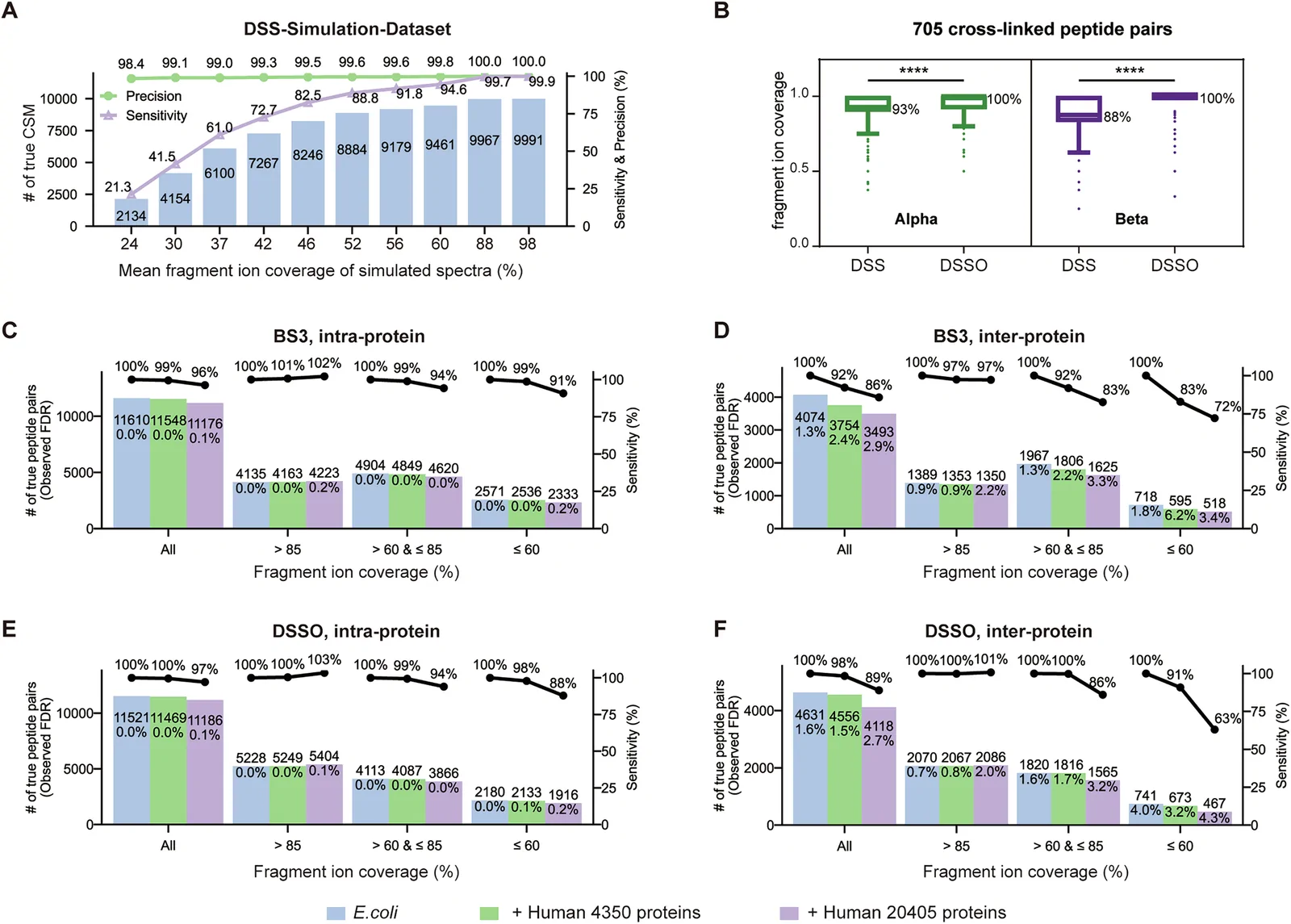
Differences between DSS and DSSO in fragment ion coverage and their effect on identification sensitivity. (**A**) Sensitivity of identification as a function of sequence coverage of fragment ions on simulated MS2 of DSS cross-links when searched against 100 human proteins. (**B**) Statistics on fragment ion coverage of synthetic peptides cross-linked with DSS or DSSO, based on experimental MS2 spectra (PXD071417). All peptide sequences are listed in Appendix Table S1. Data are represented as Tukey box plots. The center line denotes the median (50th percentile), and the box bounds represent the 25th and 75th percentiles. The whiskers extend to the most extreme data points that are no more than 1.5× the interquartile range from the box edges. Data points beyond the whiskers are plotted individually as outliers. Both α and β peptides exhibited statistically significant differences in coverage between the cross-linkers (Mann–Whitney *U* test; α peptide *P* = 2.45e-44, β peptide *P* = 1.36e-265; *n* = 3514 and *n* = 3486 for DSS and DSSO, respectively). (**C**–**F**) Re-identification of cross-links of *E. coli* proteins with zero, 4350, or 20,405 human protein sequences appended to the *E. coli* database and stratification by fragment ion coverage. (**C**) BS3 intra-protein cross-links, (**D**) BS3 inter-protein cross-links, (**E**) DSSO intra-protein cross-links, (**F**) DSSO inter-protein cross-links. Data source: PXD019120. Note: The actual FDR was calculated following the principle prescribed by the original study: the identified intra-fraction cross-links of *E. coli* proteins were defined as true positives whereas the inter-fraction ones were false, and cross-links with one or both peptides matching to the human entrapment database were also false; observed FDR was calculated as total number of false positives divided by the sum of the total number of false positives and the total number of true positives. Source data are available online for this figure.

To address this issue, we used 100,000 simulated spectra of DSS cross-links (see “Methods”), each with a specified fragment ion coverage in the range of 0 to 100%. Of note, between the fragment ion coverage of the α- peptide and that of the β-peptide, the lower value was used here and below. These simulated spectra were grouped by fragment ion coverage into 10 bins, each with 10,000 spectra (Fig. 5A). For each bin, the spectra were searched against a sequence database with 100 human proteins, including the target peptides. As shown, identification sensitivity increased from 21.3% to 99.9% as the mean fragment ion coverage increased from 24% to 98%. At ~60% fragment ion coverage, identification sensitivity and precision reached 94.6% and 99.8%, respectively (Fig. 5A). Searching against 20,405 human proteins showed similar results (Appendix Fig. S9). This in silico analysis demonstrates that sequence coverage by fragment ions has a big impact on identification sensitivity.

For a rigorous, side-by-side comparison of the fragment ion coverage of DSS vs DSSO cross-links, we had 705 pairs of synthetic peptides cross-linked in two ways, one with DSS and the other DSSO. These cross-links were fragmented under the same MS setting (Fusion Lumos, HCD NCE = 30). As shown in Fig. 5B, DSSO cross-links had a higher median fragment ion coverage (100% for both the α- and β-peptide) than DSS cross-links (93% for the α-peptide and 88% for the β-peptide).

We reanalyzed this dataset after binning the identified MS2 spectra by fragment ion coverage. Depending on the construction of the sequence database to be searched against, that is, whether two peptide sequences of a given pair are provided in the same protein sequence entry or not, these cross-links can be identified as either intra-protein cross-links or inter-protein cross-links. If searched as intra-protein cross-links, the sensitivity of identification remained high (above 98%) for both cross-linkers (Appendix Fig. S10A,C). In contrast, if searched as inter-protein cross-links and if a human proteome database was added to the search space, the sensitivity of identification decreased for those of ≤90% fragment ion coverage, whereas those of >90% fragment ion coverage were hardly affected, regardless of the cross-linker type (Appendix Fig. S10B,D). This shows that it is fragment ion coverage, not cleavability of cross-linker per se, that underlies the loss of identification sensitivity to search space explosion.

We further validated this result using public datasets of high-complexity samples—*E. coli* lysates cross-linked with BS3 or DSSO (Lenz et al, 2021). Cross-linking products of BS3 and DSS are identical. As reported (Kolbowski et al, 2022), the BS3-linked peptide pairs have a slightly lower median fragment ion coverage (91.7% for the α-peptide and 80.0% for the β-peptide) than the DSSO-linked ones (100.0% for the α- peptide and 83.3% for the β-peptide) (Appendix Fig. S11). As shown (Fig. 5C–F), for intra-protein cross-links of either BS3 (Fig. 5C) or DSSO (Fig. 5E), the sensitivity of re-identification remained above 98% for spectra with fragment ion coverage above 85%. For those with fragment ion coverage no more than 60%, adding tens of thousands of human protein sequences to the search space reduced their re-identification sensitivity to 91% or lower (Fig. 5C,E). Such a negative impact was most evident for inter-protein cross-links of either BS3 (re-identification rate down to 72%, Fig. 5D) or DSSO (re-identification rate down to 63%, Fig. 5F), if their fragment ion coverage was not higher than 60%. This accounts for much of the sensitivity loss when the MS2 spectra of inter-protein cross-links were searched undivided against a sequence database containing tens of thousands of proteins (Fig. 5D,F).

Collectively, the above results indicate that due to a higher sequence coverage by fragment ions, MS2 spectra of DSSO cross-links are less likely to lose identification as the search space expands.

## Discussion

Cross-linking mass spectrometry as an approach to protein interactome mapping is being greeted with exuberant enthusiasm. A sample for interactome mapping is extremely complex. It contains large amounts of unwanted linear peptides and mono-linked peptides, and small amounts of cross-linked peptides. Of the cross-linked peptides, most are intra-protein cross-links, which are not informative for interactome mapping. How to identify as many inter-protein cross-links as possible in a sea of other unwanted peptides is a formidable challenge.

To tackle the challenge, cross-linkers with enrichment functions were developed (Chen et al, 2025; Jiang et al, 2022; Steigenberger et al, 2019a; Wheat et al, 2021). Cleavable cross-linkers, including DSSO and its derivatives, which generate signature peptide ions believed to be beneficial to interactome mapping, were also tried in conjunction with extensive fractionation (Bogdanow et al, 2023; O’Reilly et al, 2020). However, Lars Kolbowski and colleagues have shown that for DSSO cross-links, their signature peptide ions, which are absent in 2–10% of the CSMs, provide no advantage to cross-link identification (Kolbowski et al, 2022). The same study shows that DSSO cross-links have higher fragment ion coverage than DSS cross-links, and this is the primary advantage of DSSO.

In this study, we show that incomplete sequence coverage by fragment ions is a major reason why the sensitivity of identification of inter-protein cross-links deteriorates substantially when MS2 spectra are searched against a large database containing thousands of proteins or even an entire human proteome. This represents a considerable obstacle. Recognizing this problem and solving it will be a big step forward for interactome mapping.

Incomplete sequence coverage is not an imperfection owned by DSS cross-links; it is shared by DSSO cross-links (Fig. 5; Appendix Fig. S10). Although DSSO cross-links collectively have higher sequence coverage than DSS cross-links by up to 12% in median value, there is a significant fraction of low-sequence coverage ones to cause sensitivity loss in cross-link search at a proteome scale (Figs. 4 and  5). Fragment ion coverage needs to reach 85% or above to maintain identification sensitivity for interactome mapping (Fig. 5).

We think that neither DSS nor DSSO suffices for interactome mapping. It is necessary to develop cross-linkers that would allow separation of inter-protein cross-links away from intra-protein cross-links, mono-links, and unmodified peptides. Before such ideal cross-linkers become available, improvements can be made to existing cross-linkers. Concerning DSS, since it produces more protein cross-links than DSSO (Figs. 1 and 2), increasing fragment ion coverage may be a priority. For instance, stepped HCD could be tested to see if it fragments DSS cross-links better than a single HCD energy (NCE 30%, presently). Concerning DSSO, its spacer arm is relatively short and rigid. The latter is due to the S = O bond, which makes DSSO cleavable, so it cannot be disowned, but the spacer arm of DSSO may be extended to access more cross-linkable sites.

## Methods

**Table:** Reagents and Tools Table

Reagent/resource	Reference or source	Identifier or catalog number
**Chemicals, enzymes, and other reagents**		
DSS (Disuccinimidyl suberate)	Thermo Scientific	A39267
BS3 (bis(sulfosuccinimidyl) suberate)	Thermo Scientific	A39266
DSSO (disuccinimidyl sulfoxide)	Thermo Scientific	A33545
BSMEG (bis(succinimidyl) mono(ethylene glycol))	BroadPharm Inc.	BP-21832
DSH (disuccinimidyl heptanoate)	BOC Sciences	74648-14-9
Urea	Sigma-Aldrich	U0631
TCEP-HCl	Sigma-Aldrich	75259
Trypsin (Gold)	Promega	V5280
**Software**		
pLink 3 (3.0.16)	https://github.com/pFindStudio/pLink3	
Scout (1.6.3)	https://github.com/diogobor/Scout	
pLink3-PepIon	https://github.com/daheitu/filtering-enabled-version-of-pLink	
pLink3-doublet	https://github.com/daheitu/filtering-enabled-version-of-pLink	
**Other**		

### Chemicals and reagents

The primary cross-linkers used in this study—disuccinimidyl suberate (DSS), bis(sulfosuccinimidyl) suberate (BS3), and disuccinimidyl sulfoxide (DSSO)—were purchased from Thermo Fisher Scientific (Waltham, MA, USA). The additional cross-linkers used for physicochemical comparisons, bis(succinimidyl) mono(ethylene glycol) (BSMEG, CAS: 65869-64-9) and disuccinimidyl heptanoate (DSH, CAS: 74648-14-9), were obtained from BroadPharm Inc. (San Diego, CA, USA) and BOC Sciences (Shirley, NY, USA), respectively.

All other standard reagents for sample preparation and enzymatic hydrolysis were of high purity. Urea, tris(2-carboxyethyl) phosphine (TCEP), and iodoacetamide (IAA) were purchased from MedChemExpress (Monmouth Junction, NJ, USA). Acetonitrile (ACN), formic acid (FA), trifluoroacetic acid (TFA), and Tris buffer were obtained from Thermo Fisher Scientific.

### Preparation of *E. coli* cell lysate

*E. coli* strain MG1655 cells were cultured in M9 medium for ∼12 h until OD_600_ reached 0.6–0.8. *E. coli* cells were pelleted at 4000× *g* for 20 min at 4 °C and stored at −80 °C. The frozen cell pellet was resuspended in 500 μL of ice-cold extraction buffer (50 mM HEPES, 150 mM NaCl, pH 8.2, supplemented with 1 mM PMSF and 1× complete protease inhibitor cocktail). Then, 0.5 mL of sterile, pre-chilled glass beads (0.1 mm diameter) was added, and the cells were lysed using a FastPrep homogenizer (6.5 m/s for 20 s per cycle, with 3 total cycles). The lysate was centrifuged at 14,000 × *g* (4 °C) for 30 min. The supernatant was transferred to a fresh tube. The protein concentration was measured using the BCA assay according to the manufacturer’s protocol.

### Cross-linking of DSS, BSMEG, DSH, and DSSO

Protein samples (BSA, lactoferrin, lysozyme, aldolase, GST, PUD 1/2, CNGP, UtpA, yeast ribosome, and *E. coli* lysate) were diluted to 0.75 mg/mL with HEPES buffer (50 mM HEPES, 150 mM NaCl, and pH 7.5). Cross-linkers, including DSS, BSMEG, DSH, and DSSO, were diluted with the same HEPES buffer. The cross-linking reactions were used with 15 μg protein samples and 1 mM cross-linker for 45 min and quenched by the addition of 20 mM NH_4_HCO_3_. Then proteins were precipitated with 6-fold volume of pre-chilled acetone at −20 °C overnight.

### Trypsin digestion of cross-linked protein samples

To each sample, the precipitated proteins were resuspended with 10 μL 8 M urea (100 mM Tris, pH 8.5) and sonicated for 10 min to enhance solubility. Tris(2-carboxyethyl)phosphine (TCEP) was added to a final concentration of 5 mM, and mixtures were incubated at RT for 20 min. Next, iodoacetamide (IAA) was added to a final concentration of 10 mM, and the samples were incubated at RT for 15 min in the dark. Samples were added with 30 μL 20 mM methylamine (100 mM Tris, 1 mM CaCl_2_, pH 8.5). For digestion, trypsin (1:50 enzyme to protein concentration) was added, followed by overnight incubation at 37 °C. Finally, the resulting peptides were acidified by adding formic acid (FA) to a final concentration of 5% to terminate digestion. Peptides were desalted using desalting columns (Pierce™ C18 Spin Column) following the manufacturer’s protocol. The elution samples were concentrated in a SpeedVac centrifuge and subsequently reconstituted in 1% formic acid.

### Preparation of samples for interactome mapping from HEK293T cells

HEK293T cells were lysed in PBS buffer, 1 mL (1 mg/mL) lysate was treated with 1 mM of DSSO or BS3 for 45 min at room temperature, precipitated with 25% Trichloroacetic Acid (TCA), and resuspended for trypsin digestion as described above. The resulting peptide mixtures were separated into 60 fractions by SCX chromatography (PolySULFOETHYL A column, 60-min gradient from 100% Buffer A (10 mM potassium phosphate, 25% acetonitrile, pH 3.0) to 100% Buffer B (0.5 M potassium phosphate, 25% acetonitrile, pH 3.0)). The latter 36 fractions for each cross-linker were analyzed by LCMS because cross-linked peptides were enriched in these fractions.

### Mass spectrometry data acquisition

The MS analysis was performed on an Orbitrap Fusion Lumos mass spectrometer (Thermo Fisher Scientific) equipped with a Nanospray Flex ion source and coupled to an EASY-nLC 1200 system (Thermo Scientific). Columns used for trapping and analytical separations were packed in-house in fritted capillaries. Trapping columns were packed in 100 μm internal diameter with C18 beads (Dr. Maisch, 10 μm particle size). Analytical columns were packed in 100 μm internal diameter with C18 beads (Dr. Maisch, 1.9-μm particle size). Elution in 75 min runs was performed with a gradient of mobile phase A (water and 0.1% formic acid) from 7 to 35% B (acetonitrile and 0.1% formic acid) over 61 min, 35% − 100% B over 5 min, and with final elution (100% B) using a further 5 min, then decreased to 10% B over 4 min at a flow rate of 350 nL/min.

Data acquisition on the Orbitrap Fusion Lumos was carried out using a data-dependent method with MS2 in the Orbitrap. MS1 scans were acquired in the Orbitrap at a resolution of 120 K with an AGC target of 5 × 10^5^, and a max injection time of 50 ms. MS2 scans were acquired in the Orbitrap at a resolution of 15 K with an AGC target of 1 × 10^5^, NCE 30 for non-cleavable cross-linked peptides and stepped HCD 21-27-33 for DSSO-linked peptide pairs, and a max injection time of 180 ms. The cycling time was set to 3 s. The dynamic exclusion time was 45 s.

### Cross-linked peptide identification

The pLink 3 (3.0.16) and Scout (v1.6.3) software were used to identify cross-linked peptides. The MS raw files were searched against the target protein database supplemented with 293 common contaminant proteins using pLink 3 software. The mass tolerance was 10 ppm for precursors and 20 ppm for fragment ions. A maximum of three missed cleavages were allowed for trypsin digestion. Carbamidomethylation of cysteine was defined as a fixed modification, and oxidation of methionine was set as a variable modification. The false discovery rate (FDR) was set to 5% at the peptide pair level.

For benchmarking the pLink3-doublet algorithm against Scout on the synthetic peptide dataset, the search parameters were kept as consistent as possible between the two tools. The precursor mass tolerance was set to 5 ppm, and the fragment mass tolerance was set to 10 ppm. The database containing Cas9 and the Crapome was obtained from the original study. For pLink3-doublet, a 1% FDR was applied at the peptide-pair level. For Scout, CSM results were filtered at 1% FDR at the CSM level, and peptide-pair results were filtered at 1% FDR at the residue-pair level because Scout does not provide quality control at the peptide-pair level.

### Overview of the pLink 3, pLink3-doublet, and pLink3-PepIon workflows

#### pLink 3 basic workflow

pLink 3 identifies cross-linked peptide pairs with a three-step strategy: (1) An open search is performed using a fragment ion index to retrieve candidate α peptides. (2) The mass of the β peptide is obtained by subtracting the mass of the candidate α peptide from the precursor ion mass. Then, a peptide mass index is used to retrieve candidate β peptides. (3) Candidate α and β peptides are combined into cross-linked peptide pairs, which subsequently undergo refined scoring to yield the final identification results.

#### pLink3-doublet workflow

For cleavable linkers, the workflow first extracts doublet peaks from MS/MS spectra using the signature delta mass produced by the cleavable linker (e.g., 31.972 Da for DSSO). Combining these doublets with the precursor mass yields candidate α/β mass pairs. The mass of the α peptide found via pLink 3’s ion index must match one of the masses in these extracted mass pairs. Consequently, given the constraint of the α peptide mass, the mass of the β peptide corresponds to the other mass in the pair.

#### pLink3-PepIon workflow

This workflow is designed for spectra from non-cleavable cross-links, aiming to simulate the mass pair filtering for α and β peptides used in cleavable cross-linking. Building upon the basic pLink 3 workflow, it checks the spectra for the presence of corresponding intact peptide ion peaks for each α peptide candidate obtained via the ion index. Candidates lacking the expected intact peptide peak are filtered out, yielding a new set of α peptide candidates constrained by peptide mass.

### Generation of simulated spectra for non-cleavable cross-links

Simulated spectra were generated with a procedure consistent with the pSimXL method (Chen et al, 2019). The occurrence probabilities and average intensities for the fragment ion types also adopt the statistics from real data reported in the pSimXL. For example, the ion occurrence probabilities are b1+ = 0.25, b2+ = 0.24, y1+ = 0.60, and y2+ = 0.33. To generate spectra with different fragment ion coverages, the ion occurrence probabilities were multiplied by different scaling factors. These scaling factors included nine levels: 15%, 20%, 25%, 30%, 35%, 40%, 45%, 50%, and 100%, plus an additional set using the higher occurrence probabilities from the pLink 2 paper (b1+ at 0.5, b2+ at 0.5, y1+ at 0.8, y2+ at 0.5), resulting in ten datasets with varying fragment ion coverage. The simulated spectra were generated using a database comprising the first 100 protein sequences from the human proteome. In silico digestion used trypsin with up to two missed cleavages. The simulated cross-linker was DSS. A total of 20,000 MS2 spectra were generated for each dataset, consisting of 10,000 cross-linked, 5000 mono-linked, and 5000 regular spectra. Peptide lengths were restricted to the range [6, 60], and peptide masses to the range [600, 6000]. MS1 tolerance was 10 ppm and MS2 tolerance was 20 ppm, with no modifications. Furthermore, intact peptide ions for both the α and β peptides were added to the spectra with a 100% occurrence probability. The mass of each such ion equaled the intact peptide mass plus 221.142 Da, corresponding to the sum of the mass of the lysine cyclic imidine form and the BS3 cross-linker.

### Calculation of precision and sensitivity in simulated datasets

A total of 10,000 simulated cross-linked spectra (denoted as x) were evaluated. The identities of the cross-linked peptide pairs for these simulated spectra were known by design: each peptide pair was created by randomly selecting two peptides from the 100-protein database, and the mass values of theoretical fragment ions were calculated for each artificially generated cross-link. A true positive match was defined as an instance where the final identification result given by the search engine matched exactly the source peptide pair that was used to generate the corresponding simulated spectrum. After applying FDR control, the total number of matches reported by the search engine was denoted as y, and the subset of true positive matches within y was denoted as z. Precision was then calculated as z/y, and sensitivity was calculated as z/x.

## Supplementary information

## Data Availability

The mass spectrometry proteomics data have been deposited to the ProteomeXchange Consortium (https://proteomecentral.proteomexchange.org) via the iProX partner repository with the dataset identifier PXD071417. The pLink3-PepIon and pLink3-doublet are available at: https://github.com/daheitu/filtering-enabled-version-of-pLink. The source data of this paper are collected in the following database record: biostudies:S-SCDT-10_1038-S44320-026-00222-9.
