# Supplementary material for: To cleave or not to cleave: a systemic evaluation of DSS versus DSSO for cross-linking mass spectrometry analysis
Source: Mol Syst Biol. 2026 Jun 9;22(9):1401–14. doi: 10.1038/s44320-026-00222-9 (PMC13538645; doi:10.1038/s44320-026-00222-9)
Supplement: Supplementary file 1 [file 44320_2026_222_MOESM1_ESM.pdf]

Appendix for: To cleave or not to cleave: a systemic evaluation of DSS versus DSSO for cross-linking mass spectrometry analysis

Table of Content

Appendix Figure S1: Optimize collision energy for DSSO-linked peptide pairs for reporter ions and backbone fragments with standard proteins......................................3

Appendix Figure S2: Optimize stepped HCD for DSSO-linked peptide pairs for reporter ions and backbone fragments with standard proteins......................................5

Appendix Figure S3: Compare the performance of pLink 3 and Scout on DSSO and DSBU datasets at CSM and peptide pair level. 6

Appendix Figure S4: Comparison of the number of mono-link PSM and peptides identified using DSS and DSSO. 7

Appendix Figure S5: Molecular dynamics simulation of stretchable ranges of DSS, DSH, BSMEG and DSSO...8

Appendix Figure S6: SDS-page analysis of DSS, DSH, BSMEG and DSSO cross-linked BSA and GST proteins...9

Appendix Figure S7: Statistic of KLα/β-NH_3 ions on large-scale BS3 cross-linked E. coli lysate dataset at CSM and peptide pair level. 10

Appendix Figure S8: A Venn diagram illustrating the overlap of pLink 3-doublet and Scout on DSSO and DSBU synthetic peptide datasets at CSM and peptide pair level. 11

Appendix Figure S9: Sensitivity of identification as a function of fragment ion coverage of fragment ions on simulated MS2 of DSS cross-links when search against 20405 human proteins..12

Appendix Figure S10: Re-identification of DSS and DSSO-linked synthetic peptides datasets with zero, 4350, or 20405 human protein sequences appended to the intra- or inter-peptide database and stratification by fragment ion coverage. 13

Appendix Figure S11: Violin plot to compare the fragment ion coverage of BS3 and DSSO cross-linked peptides from *E. coli* lysate...14

Appendix Table S1: The sequence of synthetic peptides...15

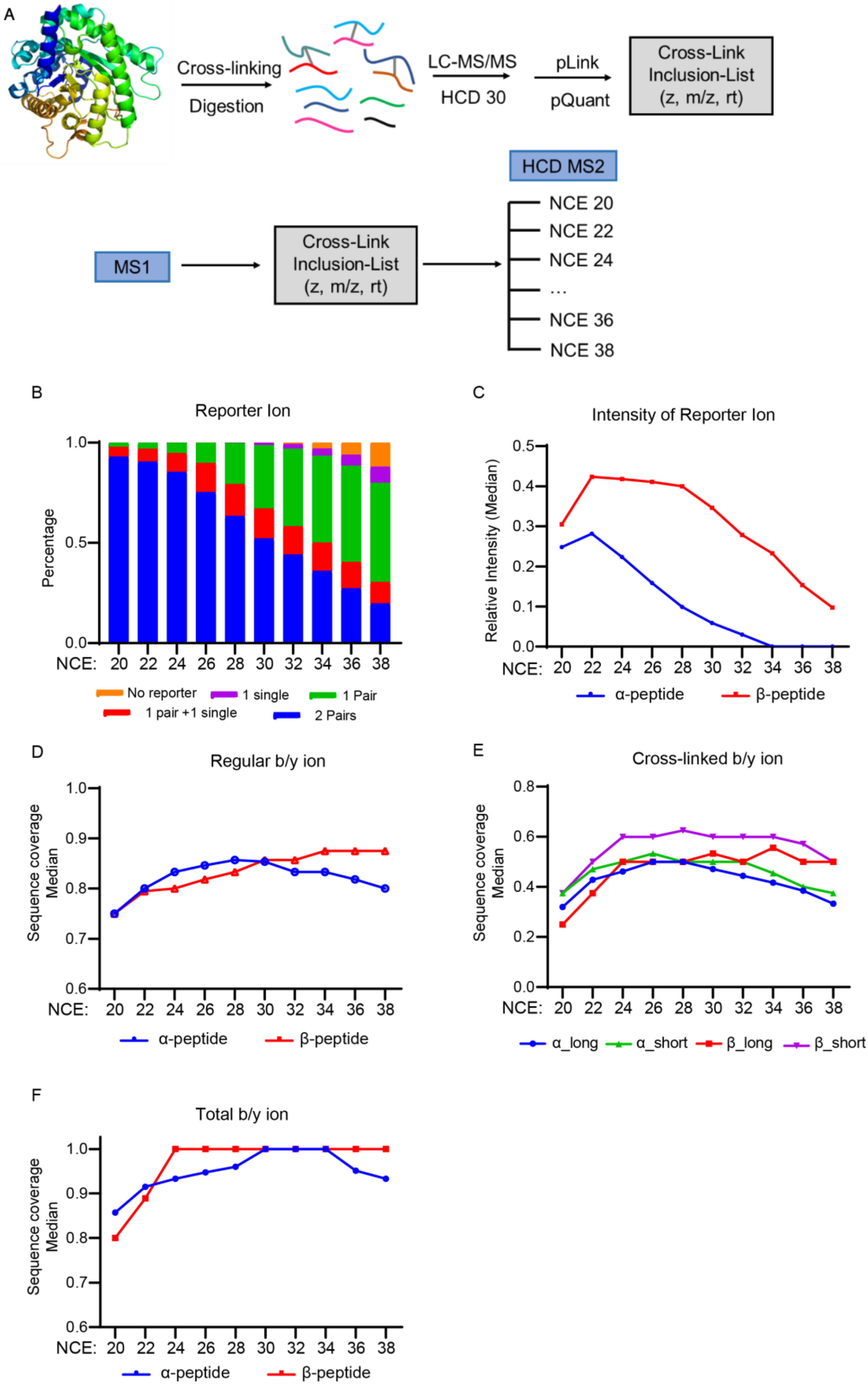

A
Cross-linking
Digestion
LC-MS/MS
HCD 30
pLink
pQuant
Cross-Link
Inclusion-List
(z, m/z, rt)
HCD MS2
MS1
Cross-Link
Inclusion-List
(z, m/z, rt)
NCE 20
NCE 22
NCE 24
…
NCE 36
NCE 38
B
Reporter Ion
Percentage
1.0
0.5
0.0
NCE: 20 22 24 26 28 30 32 34 36 38
No reporter
1 single
1 Pair
1 pair +1 single
2 Pairs
C
Intensity of Reporter Ion
Relative Intensity (Median)
0.5
0.4
0.3
0.2
0.1
0.0
NCE: 20 22 24 26 28 30 32 34 36 38
α-peptide
β-peptide
D
Regular b/y ion
Sequence coverage
Median
1.0
0.9
0.8
0.7
0.6
NCE: 20 22 24 26 28 30 32 34 36 38
α-peptide
β-peptide
E
Cross-linked b/y ion
Sequence coverage
Median
0.8
0.6
0.4
0.2
0.0
NCE: 20 22 24 26 28 30 32 34 36 38
α_long
α_short
β_long
β_short
F
Total b/y ion
Sequence coverage
Median
1.0
0.9
0.8
0.7
0.6
NCE: 20 22 24 26 28 30 32 34 36 38
α-peptide
β-peptide

Appendix Figure S1: Optimize collision energy for DSSO-linked peptide pairs for reporter ions and backbone fragments with standard proteins BSA, lactoferrin, lysozyme, aldolase.

(A) Schematic of the two-step workflow for NCE optimization. First, a mixture of four proteins was cross-linked with DSSO, digested, and analyzed via LC-MS/MS with HCD fragmentation (NCE=30). The resulting data was processed with pLink and pQuant to generate an inclusion list containing the mass-to-charge ratio (m/z), charge state (z), and retention time (rt) of confidently identified cross-linked peptides. Second, a targeted mass spectrometry method was employed to re-acquire MS2 spectra for these precursors, systematically varying the HCD NCE from 20 to 38 in steps. (B) Distribution of reporter ion pair completeness across the tested NCE range. Bar plots show the proportion of MS2 spectra containing reporter ions for both α and β peptides, for only one peptide, or for neither. (C) The median of relative intensity of α and β peptide reporter ions under each stepped NCE. (D-F) The median of sequence coverage of regular b/y ions (D), cross-linked b/y ions (E) and total b/y ions (F) of α and β peptide under each stepped NCE.

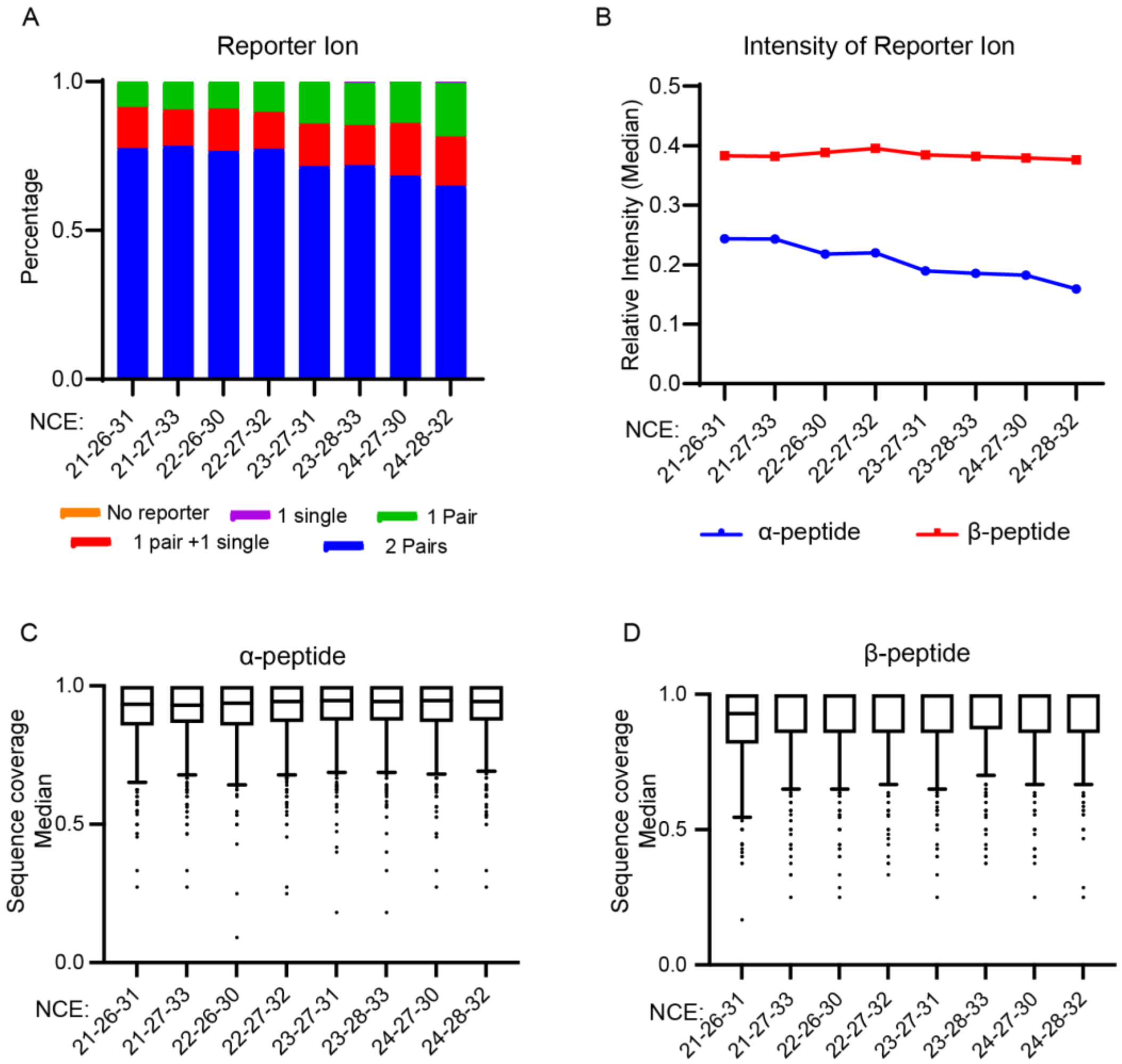

Appendix Figure S2: Optimize stepped HCD for DSSO-linked peptide pairs for reporter ions and backbone fragments with standard proteins BSA, lactoferrin, lysozyme, aldolase. (A) The proportion of doublet of α and β peptide under each stepped NCE, two pairs, one pair plus one single, one pair. (B) The median of relative intensity of α and β peptide reporter ions under each stepped NCE. (C-D) The median of fragment ion coverage of α peptide (C) and β peptide (D) under each stepped NCE.

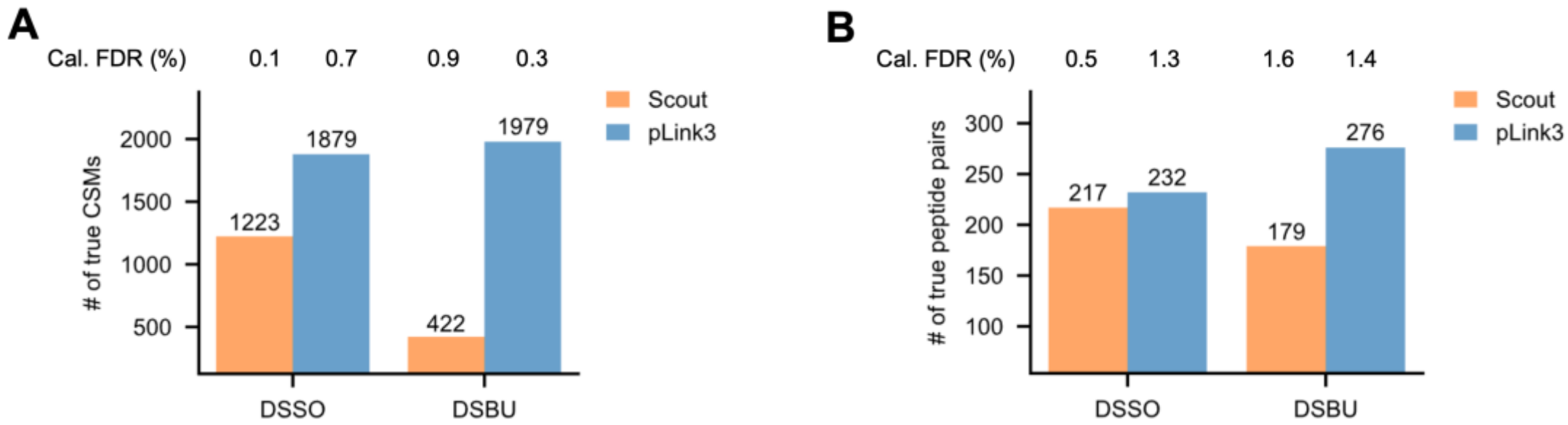

Appendix Figure S3: Compare the performance of pLink 3 and Scout on DSSO and DSBU datasets at CSM (A) and peptide pair (B) level. Data source: DSSO and DSBU cross-linked 95 synthetic peptides from Mechtler Lab, PXD014337.

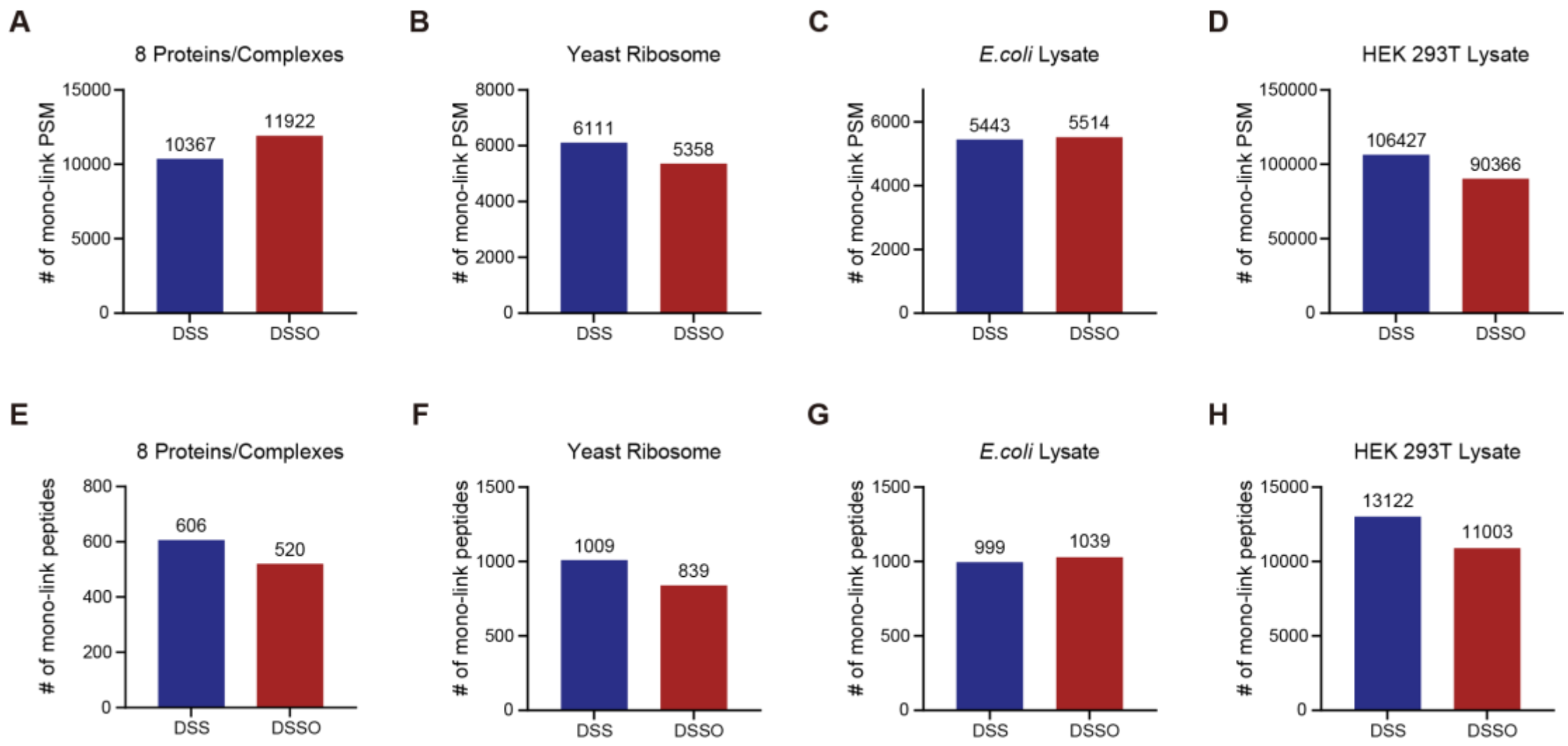

Appendix Figure S4: Comparison of the number of mono-link PSM and peptides identified using DSS and DSSO. Data are shown for (A, E) the eight purified proteins and complexes, (B, F) yeast ribosome, (C, G) *E. coli* lysate, and (D, H) HEK293T lysate.

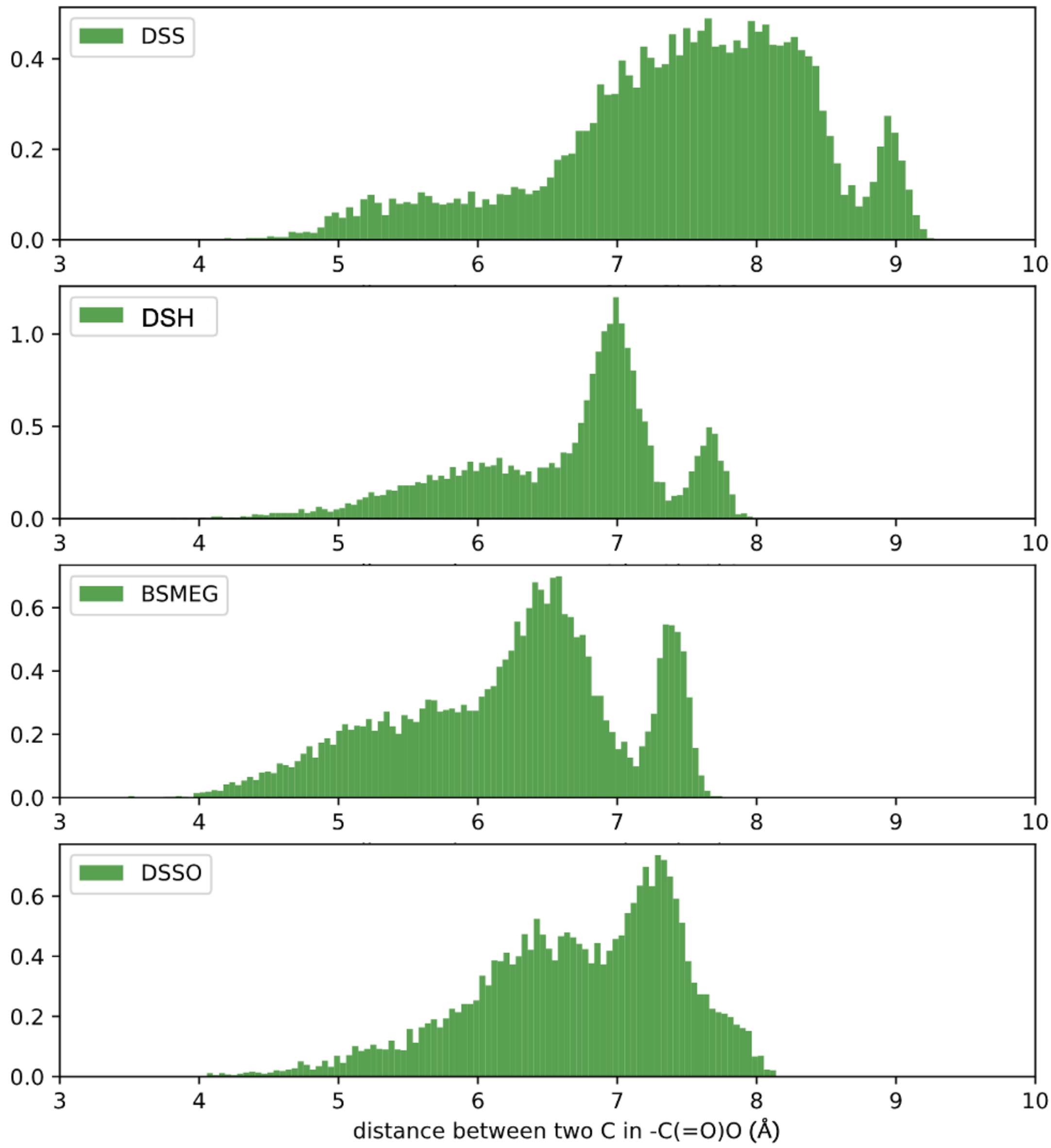

Appendix Figure S5: Molecular dynamics simulation of stretchable ranges of DSS, DSH, BSMEG and DSSO. The x-axis showed the distance between two carbon atoms in -C(=O)-O, the y-axis showed the frequency.

A

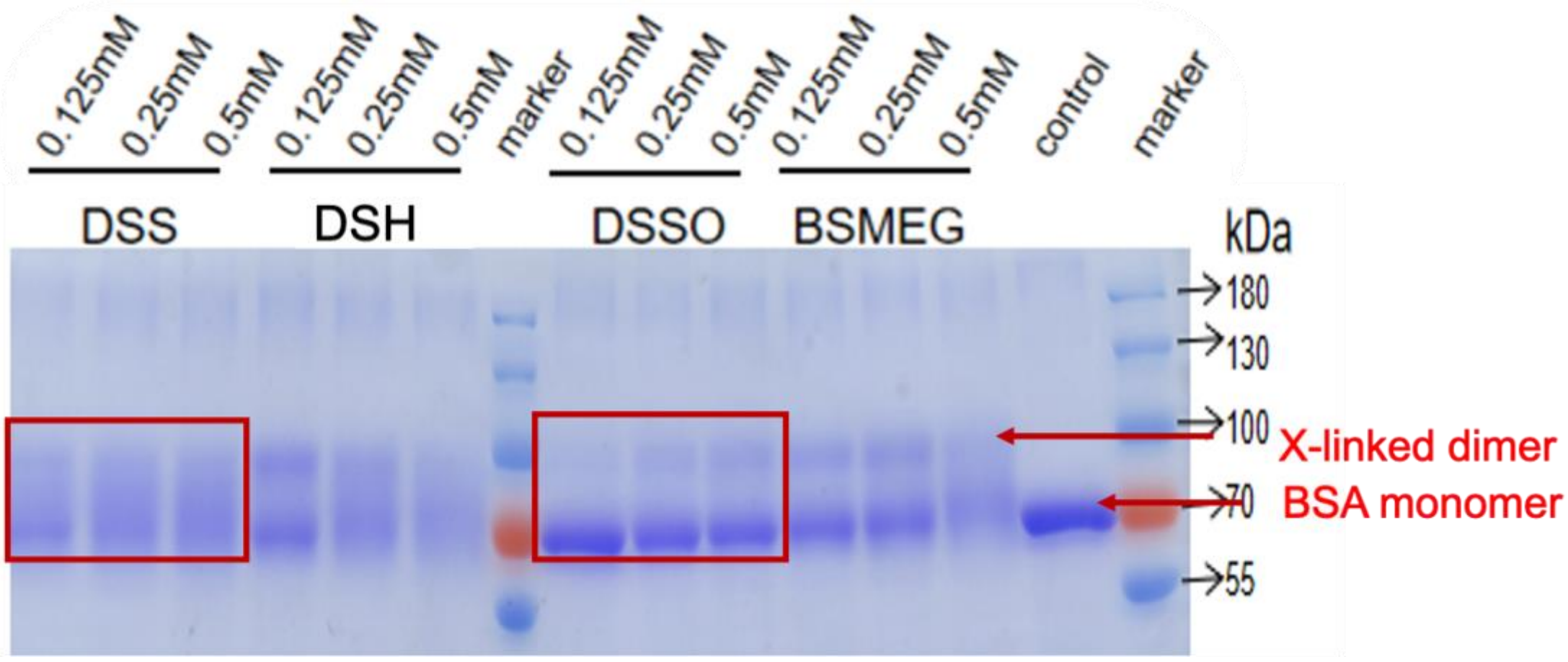

B

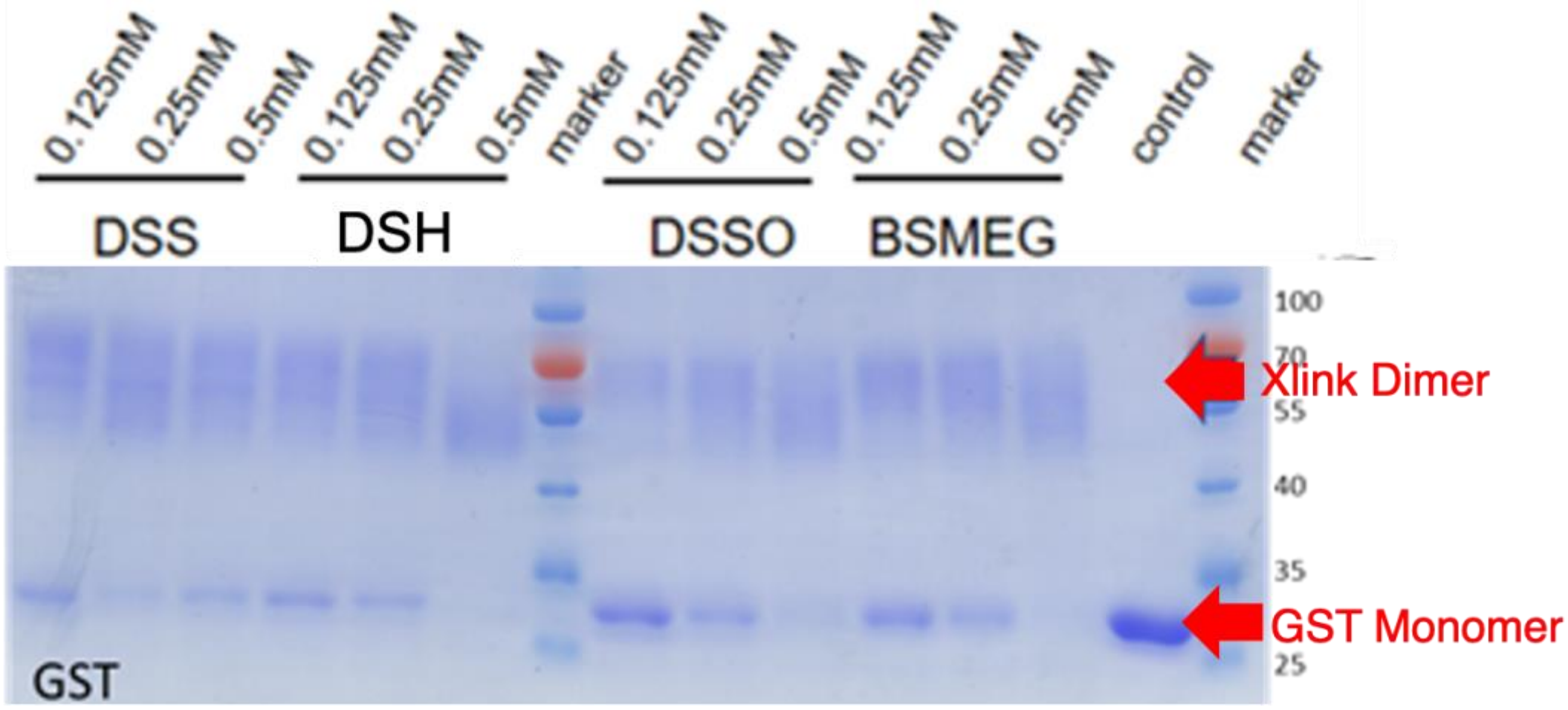

Appendix Figure S6: SDS-page analysis of DSS, DSH, BSMEG and DSSO cross-linked BSA (A) and GST (B) proteins.

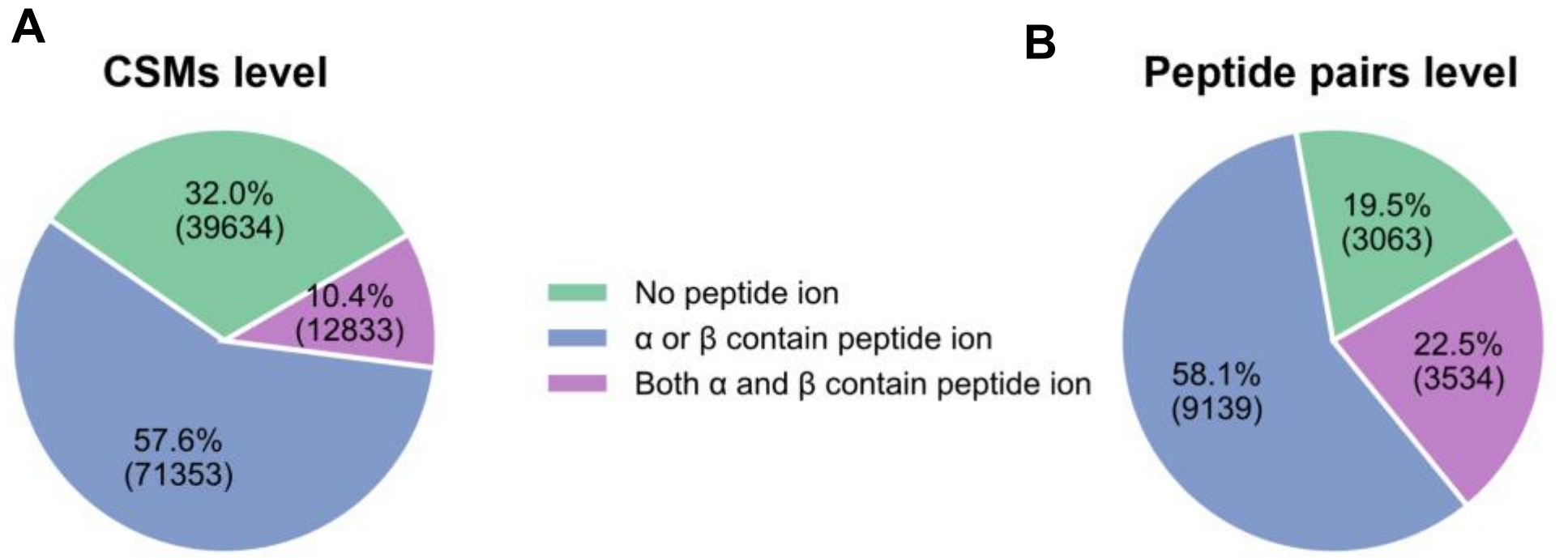

Appendix Figure S7: Statistic of KLα/β-NH_3 ions on large-scale BS3 cross-linked *E. coli* lysate dataset (PXD019120) at CSM (A) and peptide pair level (B).

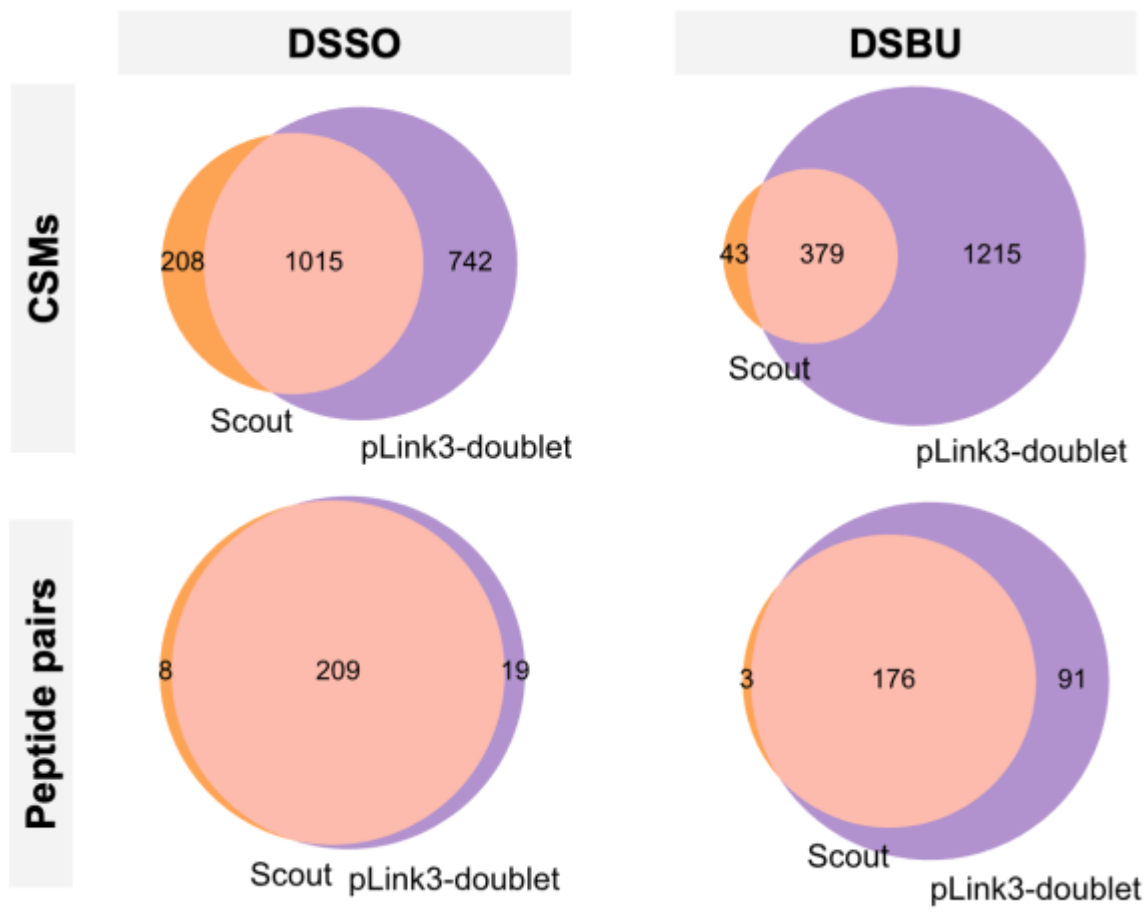

Appendix Figure S8: A Venn diagram illustrating the overlap of pLink 3-doublet and Scout on DSSO and DSBU synthetic peptide datasets at CSM and peptide pair level. Data source: DSSO and DSBU cross-linked 95 synthetic peptides from Mechtler Lab, PXD014337.

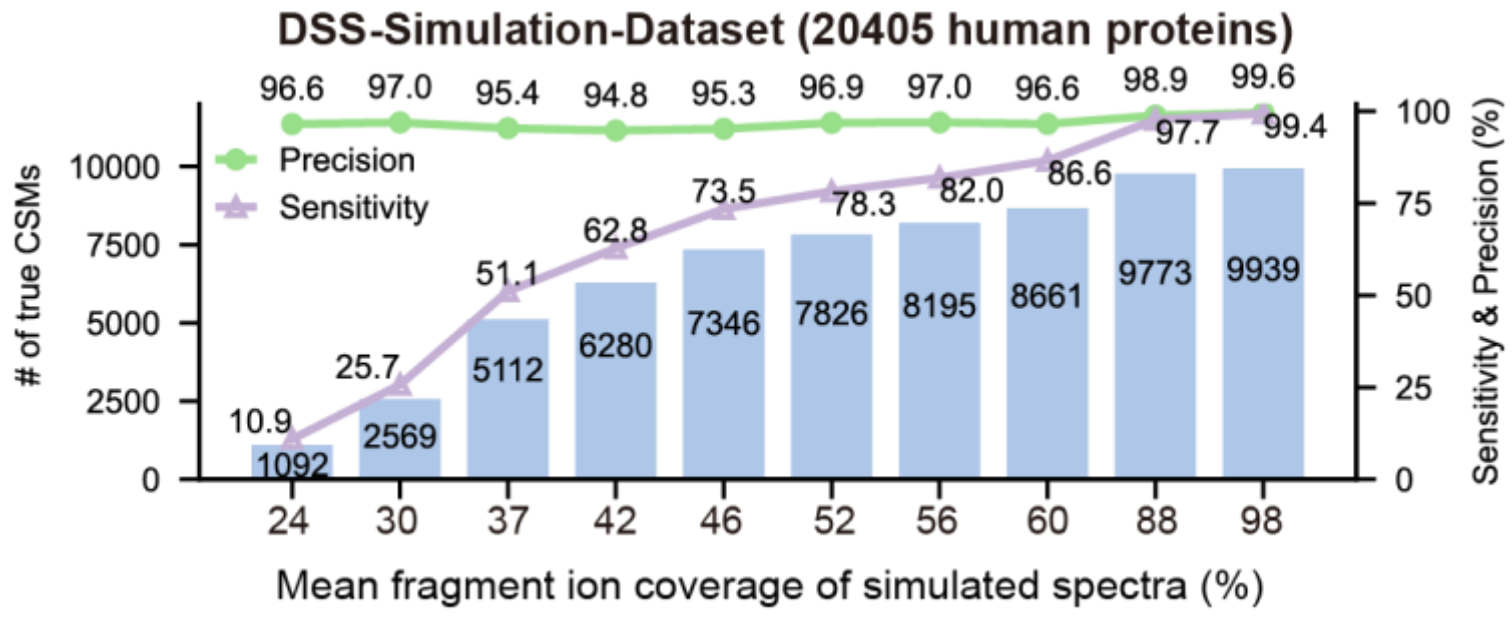

Appendix Figure S9: Sensitivity of identification as a function of fragment ion coverage of fragment ions on simulated MS2 of DSS cross-links when search against 20405 human proteins.

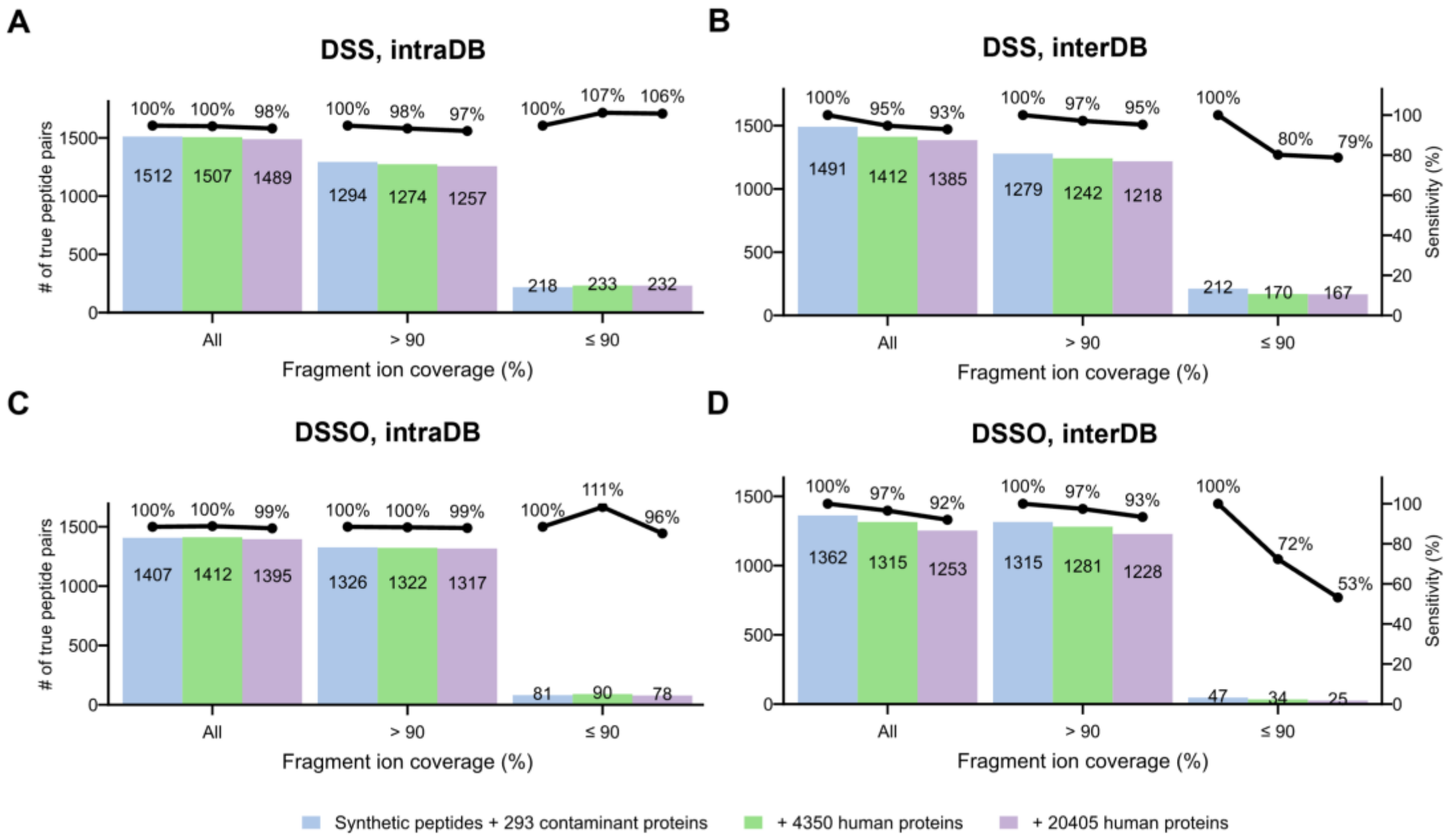

Appendix Figure S10: Re-identification of DSS and DSSO-linked synthetic peptides datasets with zero, 4350, or 20405 human protein sequences appended to the intra- or inter-peptide database with 293 contaminant proteins and stratification by fragment ion coverage. (A) DSS intra-DB search, (B) DSS inter-DB search, (C) DSSO intra-DB search, (D) DSSO inter-DB search. Data source: DSS and DSSO cross-linked synthetic peptides from our Lab, PXD071417.

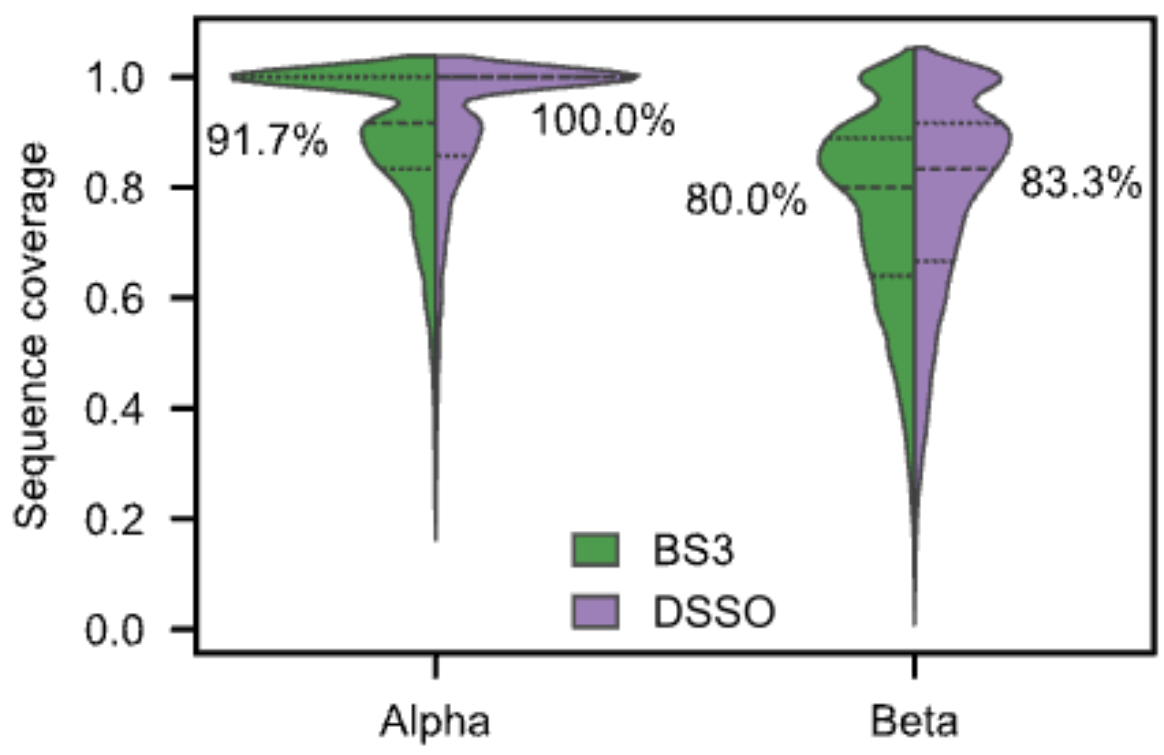

Appendix Figure S11: Violin plot to compare the fragment ion coverage of BS3 and DSSO cross-linked peptides from *E. coli* lysate (PXD019120). The alpha (longer)-peptide and beta(shorter) peptide were separated. The median of each was indicated beside.

Appendix Table S1: The sequence of synthetic peptides

Order	Name	Sequence	Length
CV1	DR-14	D(I/P/Y)ELVKAELAQ(A/W)VR	14
CV2	VR-11	V(Y/R)DKAG(T/S/D)APLK	11
CV3	FR-9	F(Y/R)QQKN(T/S/D)LR	9
CV4	AK-8	A(T/Q/G)KEIA(N/F)K	8
CV5	IR-9	IE(A/L/R)QSKL(G/W)R	9
CV6	AR-7	(A/L/R)SQK(G/W)PR	7
CV7	SK-15	S(I/P/Y)HSDVPKSGVTE(A/W)K	15
CV8	GK-13	GT(I/P/Y)FVDKQGSE(A/W)K	13
CV9	DK-8	D(T/Q/G)VIKP(N/F)K	8
CV10	ER-7	EA(T/Q/G)KL(N/F)R	7
CF1	IK-10	IKFEMEQNLK	10
CF2	DR-8	DLQTKVGR	8
CF3	AR-7	ASYKLDR	7
CF4	HK-8	HLPVKGWK	8
CF5	VR-7	VDLKHQR	7
CF6	QK-9	QGTAKFNLK	9
