# Supplementary material for: To cleave or not to cleave: a systemic evaluation of DSS versus DSSO for cross-linking mass spectrometry analysis
Source: Mol Syst Biol. 2026 Jun 9;22(9):1401–14. doi: 10.1038/s44320-026-00222-9 (PMC13538645; doi:10.1038/s44320-026-00222-9)
Supplement: Supplementary file 2 [file 44320_2026_222_MOESM2_ESM.pdf]

molecular systems biology

To cleave or not to cleave: a systemic evaluation of DSS versus DSSO for cross-linking mass spectrometry analysis

Yong Cao, Peng-Zhi Mao, Qing-Cui Wu, Zhen-Lin Chen, Jin-Cai Yang, Yue Zhao, Adalet Memetimin, Hao Chi, Xiangbing Qi, Si-Min He, and Mengqiu Dong

Corresponding author(s): Mengqiu Dong (dongmengqiu@nibs.ac.cn), Si-Min He (smhe@ict.ac.cn), Xiangbing Qi (qixiangbing@nibs.ac.cn)

Review Timeline:

Submission Date:	4th Mar 26
Editorial Decision:	19th Mar 26
Revision Received:	11th May 26
Editorial Decision:	21st May 26
Revision Received:	23rd May 26
Accepted:	27th May 26

Editor: Jingyi Hou

Transaction Report:

(Note: With the exception of the correction of typographical or spelling errors that could be a source of ambiguity, letters and reports are not edited. Depending on transfer agreements, referee reports obtained elsewhere may or may not be included in this compilation. Referee reports are anonymous unless the Referee chooses to sign their reports.)

20th Mar 2026

Manuscript Number: MSB-2026-13676
Title: To cleave or not to cleave: a systemic evaluation of DSS versus DSSO for cross-linking mass spectrometry analysis
Author: Yong Cao
Peng-Zhi Mao
Qing-Cui Wu
Zhen-Lin Chen
Jin-Cai Yang
Yue Zhao
Adalet Memetimin
Hao Chi
Xiangbing Qi
Si-Min He
Mengqiu Dong

Dear Dr Dong,

Thank you for submitting your work to Molecular Systems Biology. We have now heard back from the three reviewers who agreed to evaluate your manuscript. As you will see from the reports below, the reviewers acknowledge the interest of the study. They raise, however, a series of concerns, which we would ask you to address in a major revision.

While Reviewers #2 and #3 were generally supportive, Reviewer #1 raised concerns regarding the study's conceptual novelty and experimental design. We ask that you clearly highlight the novelty of your work in the context of existing literature, respond to Reviewer #1's concerns about differences in spacer length, and carefully address the points raised by Reviewer #2. Regarding the comparison with DSBSO suggested by Reviewer #1, we encourage you to consider including some levels of experimental data or, at a minimum, discuss its potential relevance in writing.

All other issues raised by the reviewers need to be satisfactorily addressed as well. As you may already know, our editorial policy allows in principle a single round of major revision so it is essential to provide responses to the reviewers' comments that are as complete as possible. Please feel free to contact me in case you would like to discuss in further detail any of the issues raised by the reviewers.

On a more editorial level, we would ask you to address the following issues:

- Please provide a .docx formatted version of the manuscript text (including legends for main figures, EV figures and tables). Please make sure that the changes are highlighted to be clearly visible.

- Please provide individual production quality figure files as .eps, .tif, .jpg (one file per figure). When assembling figures, please refer to our figure preparation guideline in order to ensure proper formatting and readability in print as well as on screen:
https://media.springernature.com/original/springer-cms/rest/v1/content/27825798/data/v1

-Please provide a .docx formatted letter INCLUDING the reviewers' reports and your detailed point-by-point responses to their comments. As part of the EMBO Press transparent editorial process, the point-by-point response is part of the Review Process File (RPF), which will be published alongside your paper.

-Please note that all corresponding authors are required to supply an ORCID ID for their name upon submission of a revised manuscript.

-We replaced Supplementary Information with Expanded View (EV) Figures and Tables that are collapsible/expandable online. A maximum of 5 EV Figures can be typeset. EV Figures should be cited as 'Figure EV1, Figure EV2" etc... in the text and their respective legends should be included in the main text after the legends of regular figures.

Additional Tables/Datasets should be labeled and referred to as Table EV1, Dataset EV1, etc. Legends have to be provided in a separate tab in case of .xls files. Alternatively, the legend can be supplied as a separate text file (README) and zipped together with the Table/Dataset file.

For the figures and tables that you do NOT wish to display as Expanded View figures, they should be bundled together with their legends in a single PDF file called *Appendix*, which should start with a short Table of Content. Each legend should be below the corresponding Figure/Table in the Appendix. Appendix figures and tables should be referred to in the main text as: "Appendix Figure S1, Appendix Figure S2, Appendix Table S1" etc. See detailed instructions regarding expanded view here:

https://link.springer.com/journal/44320/submission-guidelines#cms-Expanded-View-data.

-Before submitting your revision, primary datasets (and computer code, where appropriate) produced in this study need to be deposited in an appropriate public database.
Please remember to provide a reviewer password if the datasets are not yet public.
The accession numbers and database should be listed in a formal "Data Availability" section (placed after Materials & Method) that follows the model below (see also https://link.springer.com/partners/embo-press/editorial-policies#Data%20availability%20statement). Please note that the Data Availability Section is restricted to new primary data that are part of this study.
Data availability
The datasets (and computer code) produced in this study are available in the following databases:
- RNA-Seq data: Gene Expression Omnibus GSE46843 (https://www.ncbi.nlm.nih.gov/geo/query/acc.cgi?acc=GSE46843)
- [data type]: [name of the resource] [accession number/identifier/doi] ([URL or identifiers.org/DATABASE:ACCESSION])
*** Note - All links should resolve to a page where the data can be accessed. ***

- At EMBO Press we ask authors to provide source data for the main manuscript figures. You will receive a separate email with instructions for providing source data with your revised manuscript, including how to upload and organize the files.
Additional information on source data and instruction on how to label the files are available < https://link.springer.com/journal/44320/submission-guidelines#cms-Source-data >

- Our journal encourages inclusion of *data citations in the reference list* to directly cite datasets that were re-used and obtained from public databases. Data citations in the article text are distinct from normal bibliographical citations and should directly link to the database records from which the data can be accessed. In the main text, data citations are formatted as follows: "Data ref: Smith et al, 2001". In the Reference list, data citations must be labeled with "[DATASET]". A data reference must provide the database name, accession number/identifiers and a resolvable link to the landing page from which the data can be accessed at the end of the reference. Further instructions are available at < https://link.springer.com/journal/44320/submission-guidelines#cms-Reference-guidelines >.

- We updated our journal's competing interests policy in January 2022 and request authors to consider both actual and perceived competing interests. Please review the policy (https://link.springer.com/partners/embo-press/editorial-policies#Competing%20interest%20disclosures) and update your competing interests if necessary.
Please use the heading "Disclosure statement and competing interests".

- All Materials and Methods need to be described in the main text using our 'Structured Methods' format. According to this format, the Methods section includes a Reagents and Tools Table (listing key reagents, experimental models, software and relevant equipment and including their sources and relevant identifiers) followed by a Methods and Protocols section describing the methods, ideally using a step-by-step protocol format. The aim is to facilitate adoption of the methodologies across labs.

Please download and fill our Reagents and Tools Table template (.docx), which you can find in our author guidelines: https://link.springer.com/journal/44320/submission-guidelines#structuredmethods.

When submitting your revised manuscript, please do not include the Reagents and Tools Table in the Methods section of the manuscript but upload it as a separate file choosing the file type "Reagent Table".

-Regarding data quantification:
Please ensure to specify the name of the statistical test used to generate error bars and P values, the number (n) of independent experiments (please specify technical or biological replicates) underlying each data point and the test used to calculate p-values in each figure legend. Discussion of statistical methodology can be reported in the materials and methods section, but figure legends should contain a basic description of n, P and the test applied.
Graphs must include a description of the bars and the error bars (s.d., s.e.m.).
Please also include scale bars in all microscopy images.

- Please provide a "standfirst text" summarizing the study in one or two sentences (approximately 250 characters, including space), three to four "bullet points" highlighting the main findings and a " visual abstract" (550px width and 400-600 px height, PNG format) to highlight the paper on our homepage.
Please refer to published papers for examples.

When you resubmit your manuscript, please download our CHECKLIST (https://media.springernature.com/original/springer-cms/rest/v1/content/27825796/data/v1) and include the completed form in your submission.
Please note that the Author Checklist will be published alongside the paper as part of the transparent process .

If you feel you can satisfactorily deal with these points and those listed by the referees, you may wish to submit a revised version of your manuscript. Please attach a covering letter giving details of the way in which you have handled each of the points raised by the referees. A revised manuscript will be once again subject to review and you probably understand that we can give you no

guarantee at this stage that the eventual outcome will be favorable.

I look forward to receiving the revised manuscript soon.

Kind regards,
Jingyi

Jingyi Hou, PhD
Senior Editor
Molecular Systems Biology

We realize that it is difficult to revise to a specific deadline. In the interest of protecting the conceptual advance provided by the work, we recommend a revision within 3 months (17th Jun 2026). Please discuss the revision progress ahead of this time with the editor if you require more time to complete the revisions. Use the link below to submit your revision:

https://msb.msubmit.net/cgi-bin/main.plex

IMPORTANT: Please read our guidelines for revised submissions: https://link.springer.com/journal/44320/submission-guidelines#cms-Revised-submissions

*** PLEASE NOTE *** As part of the EMBO Press transparent editorial process initiative (see our Editorial at https://dx.doi.org/10.1038/msb.2010.72), Molecular Systems Biology publishes online a Review Process File with each accepted manuscripts. This file will be published in conjunction with your paper and will include the anonymous referee reports, your point-by-point response and all pertinent correspondence relating to the manuscript. If you do NOT want this File to be published, please inform the editorial office at contact@molsystbiol.org within 14 days upon receipt of the present letter.

Reviewer #1:

Protein chemical cross-linking combined with mass spectrometry has become an important technique for the analysis of protein structure and protein–protein interactions.
A variety of cross-linkers are well developed. Although different cross-linkers are widely used, selecting the appropriate one for an experiment can be challenging. In this manuscript, the authors compare two widely used crosslinkers, namely DSS and DSSO. I would like to begin by explaining a few differences that are well-documented in the existing literature:

Cleavable Crosslinkers
• Mechanism: Contain a built-in "cleavable site" designed to be broken by specific stimuli (e.g., disulfide bridges by reductants, or specific MS-cleavable sites like sulfoxides).
• Best Use: Complex samples, MS-based structural proteomics (XL-MS), and ADCs requiring selective payload release.
• Advantages: Dramatically simplifies MS data analysis by separating the two crosslinked peptides (signature ions).
• Disadvantages: Can be less stable during handling than non-cleavable types.
Non-Cleavable Crosslinkers
• Mechanism: Form a permanent, stable covalent bond between molecules (e.g., DSS).
• Best Use: Studies with purified proteins, protein-protein interaction mapping when cleavage isn't required.
• Advantages: Higher structural stability, easy to handle, often lower cost.
• Disadvantages: Makes identification by MS challenging because the peptides remain tied together, creating complex mass spectra.

Key Differences Summary
• Stability: Non-cleavable is generally more stable.
• Identification: Cleavable crosslinkers provide clearer MS/MS spectra due to peptide separation.
• Flexibility: Cleavable linkers allow for on-demand removal of the crosslinking agent.

Major items:
-In my opinion, it would have been better or wiser to use a cross-linking pair with a similar spacer length. The result now differs

from the mass spectrometry fragmentation (behavior) and from the spacer length of the cross-linker. In my experience, using a cross-linking reagent with a shorter spacer yields more CSMs.

-The general question regarding this publication is how it differs from the existing literature. As an example, I would like to cite Steigenberger et al. (ASMS 2020). Or "Evaluation of cleavable crosslinking for characterization of proteoform structural differences by top-down mass spectrometry" (International Journal of Mass Spectrometry 2026). In addition, there are already some guidelines from companies that also address this topic:
- https://www.thermofisher.com/at/en/home/life-science/protein-biology/protein-labeling-crosslinking/protein-crosslinking.html?ef_id=
-Identification of MS-Cleavable and Non-Cleavable Chemically Cross-Linked Peptides Using MetaMorpheus https://pubs.acs.org/doi/10.1021/acs.jproteome.8b00141

-Regardless of this, both crosslinkers exhibit different membrane permeability and hydrophobicity (Mueller et al. Chem. Chemistry 2025)

- What I find lacking in this study is a comparison with the next reagent group, namely DSBSO, which has an affinity tag.

Minor items:
- Please correct spelling, typos, or unclear characters throughout the entire manuscript.
e.g. (cross-linkers-cleavable versus non-cleavable-remains a challenge)

- "DSS consistently yields more cross link identification s than DSSO ," This has been reported many times before and is nothing new
- "For interactome mapping of mammalian cells, neither suffices but DSSO cross-linking combined with fractionation produces better results."
Is this search engine dependent or independent? Please show data of 5 independent search strategies.
- „warheads" - A very strange expression that I had never heard before and would avoid using in a professional context

If I have misunderstood something in the manuscript or missed something, I would like to apologize to the authors in advance.

Reviewer #2:

This manuscript provides a timely and systematic evaluation of the trade-offs between non-cleavable (i.e., DSS) and cleavable (i.e., DSSO) cross-linkers. Building significantly on the work by Kolbowski et al. (Ref. 31 of the manuscript), the authors move beyond the "how" of fragmentation to provide evidence-based guidelines on "when" to use specific reagents based on sample complexity. A "crossing point" for the two cross-linkers appears to be between a bacterial-level lysate (thousands of proteins) and a mammalian-level lysate (over ten thousand proteins). The identification of the "spacer arm" volume (71.9% greater for DSS) as a primary driver for identification count in low-complexity samples is also a notable contribution. I have several critiques:

1. The authors attribute the performance gap primarily to the longer and more flexible spacer arm of DSS. To verify that this is not a result of inherent differences in chemical reactivity, the authors should provide a comparison of mono-link identifications for both reagents. This would clarify if the "warhead" efficiency is truly equivalent before concluding that the 3D "sampling volume" is the differentiator.

2. In the mammalian lysate experiments (Figure 1G), the authors switch from DSS to its sulfonated analog, BS3, to overcome solubility issues. While the cross-linked products are identical, the initial reactivity and partitioning in a complex lysate may differ between the hydrophobic DSS and the hydrophilic BS3. The authors should discuss or experimentally address how this switch might impact the validity of the comparison with DSSO. It would be even better to perform a similar comparison between BS3 and DSS (see my last comment).

3. The "85% fragment ion coverage rule" is presented as a requirement for proteome-wide mapping. While the trend in Figure 5 is clear, the transition from 60% to 85% is somewhat coarse. Could the authors provide more granular data or a clearer statistical justification for why 85%-rather than 75% or 80%-is the critical threshold?

Additionally, the presentation of Figure 5 requires clarification, specifically:

> The definition of "Observed FDR" in the context of the re-identification plots (Panels C-F) should be explicitly stated in the legend.

> The method for calculating Precision and Sensitivity in the simulated datasets should be more clearly linked to the "true" matches in the simulation.

Reviewer #3:

Cao et al. revisit the topic of cleavability of chemical cross-linking reagents for structural mass spectrometry/proteomics applications, comparing the performance of the non-cleavable DSS and the cleavable DSSO in the same computational framework (pLink 3). The authors find that DSS consistently outperforms DSSO for samples of lower complexity (ranging from individual proteins up to E. coli) while DSSO performs better at the human proteome scale. The former effect is, for the first time, attributed to the increased span of the linker arm as assessed by MD simulations: Although the difference in spacer length between the two reagents appears small (approx. 1.3 Å), this results in a disproportionally higher accessible volume for DSS. At the same time, the non-cleavable DSS shows less efficient backbone cleavage, as previously also observed by others, which appears to be the most relevant effect for DSSO's improved identification rates in large search spaces.

The manuscript challenges the default assumption of some researchers in the cross-linking community that cleavable reagents are always superior to non-cleavable ones of a similar type. As a side note, it also confirms my personal experience for low-complexity samples. Nevertheless, I was surprised by the magnitude of this effect for individual proteins (Figure 2) in a side-by-side comparison with different reagents with a slightly shorter linker span.

In summary, I find this work very interesting and relevant for the cross-linking community and those researchers who use cross-linking as an auxiliary technique in their structural or systems biology projects. I have mostly minor comments for clarifications and corrections that are summarized below.

Comments:

Page 6, line 12: replications > replicate

Page 8, figure 1: In panel C, the x-axis labeling MW(Da) = 78 KD etc. needs to be corrected. For example, write MW(kDa) and then just give numbers. Make sure to distinguish between cross-links (the products) and cross-linking (the process) in the figure legend.

Page 16: "this study joins an early one ...". Please add a reference here.

Page 16: Here, the authors refer to synthetic peptides generated for this study (as opposed to re-searching a dataset of synthetic peptides from another lab, ref. 37). No further information about this is given except in Supplementary Table 1. More information needs to be added to the method section, and it should be clear throughout the manuscript (main and SI figures) which synthetic dataset is used where.

Page 19: "with extensive fractionation (refs)" Please add the references here.

Page 21: Please give the reaction time for the HEK293 cross-linking experiment.

Page 22: The specific versions of pLink 3 (and Scout) should be stated here. Is the version with the doublet functionality publicly available or was this feature added for in-house use only?

Supplementary Figure 3: Do the authors have any explanation about the large differences in CSMs between DSSO and DSBU for Scout? Did you observe any major differences in spectral properties of the identifications for your own software, pLink (for examples, in some parameters that pLink uses for scoring)?

Supplementary Figure 4: I assume that the values given on the x-axis are in Å but this should be explicitly mentioned.

Supplementary Figure 5: The figure labels say DSSC, is this the same as DSH?

Supplementary Figures, general: Please always make clear which samples correspond to the results shown, e.g. SF Figure 2 and 3.

Response to reviewers' comments

Reviewer #1:

Protein chemical cross-linking combined with mass spectrometry has become an important technique for the analysis of protein structure and protein−protein interactions.
A variety of cross-linkers are well developed. Although different cross-linkers are widely used, selecting the appropriate one for an experiment can be challenging. In this manuscript, the authors compare two widely used crosslinkers, namely DSS and DSSO. I would like to begin by explaining a few differences that are well-documented in the existing literature:

Cleavable Crosslinkers
• Mechanism: Contain a built-in "cleavable site" designed to be broken by specific stimuli (e.g., disulfide bridges by reductants, or specific MS-cleavable sites like sulfoxides).
• Best Use: Complex samples, MS-based structural proteomics (XL-MS), and ADCs requiring selective payload release.
• Advantages: Dramatically simplifies MS data analysis by separating the two crosslinked peptides (signature ions).
• Disadvantages: Can be less stable during handling than non-cleavable types.

Response 1: We thank the reviewer for his/her suggestions and comments, which we find helpful. We have improved our manuscript accordingly.

The opening part of the review report has comments on things outside the scope of this manuscript (e.g. cross-linking for ADCs) and takes a style and tone typically associated with AI-generated text (e.g., a list of bullet points). We'll address comments related to CXMS/XL-MS.

Non-Cleavable Crosslinkers
• Mechanism: Form a permanent, stable covalent bond between molecules (e.g., DSS).
• Best Use: Studies with purified proteins, protein-protein interaction mapping when cleavage isn't required.
• Advantages: Higher structural stability, easy to handle, often lower cost.
• Disadvantages: Makes identification by MS challenging because the peptides remain tied together, creating complex mass spectra.

Key Differences Summary
• Stability: Non-cleavable is generally more stable.
• Identification: Cleavable crosslinkers provide clearer MS/MS spectra due to peptide separation.

• Flexibility: Cleavable linkers allow for on-demand removal of the crosslinking agent.

Major items:

-In my opinion, it would have been better or wiser to use a cross-linking pair with a similar spacer length. The result now differs from the mass spectrometry fragmentation (behavior) and from the spacer length of the cross-linker. In my experience, using a cross-linking reagent with a shorter spacer yields more CSMs.

Response 2: The primary purpose of this manuscript is a practical one—to help users make a well-informed decision when it comes to cross-linker choice for CXMS/XL-MS analysis. We do so by conducting a head-to-head comparison between the most popular cleavable cross-linker (DSSO) and the most popular non-cleavable cross-linker (DSS), with an optimized workflow for each.

We recognize that the length of the spacer arm is one of the important factors that shape the outcome of cross-linking. The experiments in Figure 2 are dedicated to dissecting the effects of different factors, including the spacer arm length.

An additional note based on our experience—the spacer length does not scale linearly with the amount of cross-linking products identified. For instance, in our evaluation across three proteins/protein complexes, BS3 (11.4 Å)—a high-solubility version of DSS—yielded 126 cross-linked peptides pairs, whereas the shorter BS2G (7.7 Å) yielded 86 (Ding et al., *Anal. Chem.* 2016). Similarly in the present manuscript, DSS outperforms the shorter DSH cross-linker. However, EGS (16.1 Å)—a cross-linker with a longer spacer arm—did not surpass DSS (119 vs 159).

-The general question regarding this publication is how it differs from the existing literature. As an example, I would like to cite Steigenberger et al. (ASMS 2020). Or "Evaluation of cleavable crosslinking for characterization of proteoform structural differences by top-down mass spectrometry" (International Journal of Mass Spectrometry 2026). In addition, there are already some guidelines from companies that also address this topic:

Response 3: While this recent study provides valuable insights into proteoform characterization utilizing top-down mass spectrometry, our method is fundamentally different. We focus exclusively on a bottom-up proteomics workflow aimed at structural mapping and protein-protein interactions (PPIs). The computational challenges, sample complexities, and data analysis pipelines inherent to cross-linked peptides in bottom-up workflows are distinct from top-down analyses. Therefore, we believe our systematic comparison fills a specific and critical gap in current bottom-up CXMS methodology.

- https://www.thermofisher.com/at/en/home/life-science/protein-biology/protein-labeling-crosslinking/protein-crosslinking.html? ef_id=

We acknowledge that manufacturer's application notes—such as the one suggesting DSS and BS3 exhibit better performance than DSSO on standard BSA (https://www.thermofisher.com/us/en/home/life-science/protein-biology/protein-mass-spectrometry-analysis/sample-prep-mass-spectrometry/protein-interaction-crosslinking-mass-spectrometry.html)—are useful practical references. However, these are SDS-PAGE gel images not accompanied by MS analysis results. They represent isolated, anecdotal observations that are not subjected to rigorous evaluation and are not peer reviewed. This manuscript provides a systematically validated and peer-reviewed benchmark.

-Identification of MS-Cleavable and Non-Cleavable Chemically Cross-Linked Peptides Using MetaMorpheus https://pubs.acs.org/doi/10.1021/acs.jproteome.8b00141
We respectfully wish to clarify the core computational hypothesis of our work. Our objective was not simply to demonstrate that the pLink 3 search engines can identify MS-cleavable and non-cleavable cross-linked peptides. Rather, we explicitly sought to demonstrate that a targeted search strategy for MS-cleavable cross-linkers significantly enhances identification sensitivity. Therefore, we designed a controlled algorithmic experiment: we evaluated two distinct versions of the pLink 3 workflow—one utilizing the mass of the cleaved α/β peptides, the other without. This allowed us to explicitly quantify the sensitivity improvement gained by utilizing the α/β peptide mass constraint, a nuance not directly addressed by the existing search engines.

Steigenberger et al. (ASMS 2020)
We respectfully note that this publication (in the Journal of the American Society for Mass Spectrometry) is a comprehensive review article summarizing the general usage trends of cross-linkers over a ten-year period. While it is an excellent retrospective literature survey, it is not an original research paper and does not provide controlled, side-by-side evaluations. It therefore does not replace the value of strictly controlled experimental comparison as provided in our study.

Overall, we firmly believe that to the CXMS community our manuscript has a unique, practical value.

-Regardless of this, both crosslinkers exhibit different membrane permeability and hydrophobicity (Mueller et al. Chem. Chemistry 2025)
Response 4: Thanks for highlighting the work by Mueller et al. The citation should be Mueller et al. Communications Chemistry (not Chem. Chemistry). Regarding the point on membrane permeability: our study is strictly limited to in vitro cross-linking of purified proteins or lysates; it does not touch upon in-cell cross-linking or membrane permeability. We have clarified the experimental scope in the Materials and Methods section to avoid confusion.

- What I find lacking in this study is a comparison with the next reagent group, namely DSBSO, which has an affinity tag.

Response 5: The primary objective of this study was to isolate and compare the impact of the cleavable vs. non-cleavable chemical backbone on MS identification efficiency. Both DSS and DSSO, as used in this study, lack an enrichment handle. While affinity-tagged reagents like DSBSO inherently provide higher sensitivity through enrichment, including them would introduce "enrichment efficiency" as an additional confounding variable. Our study aims to provide a baseline comparison of the fragmentation chemistry itself, which remains applicable regardless of the presence or absence of an affinity tag.

Minor items:
- Please correct spelling, typos, or unclear characters throughout the entire manuscript. e.g. (cross-linkers-cleavable versus non-cleavable-remains a challenge)
Response 6: We combed through the manuscript multiple times and corrected errors we found.

- "DSS consistently yields more cross link identification s than DSSO ," This has been reported many times before and is nothing new
Response 7: We would like to know the source data based on which this conclusion is drawn. We are not aware of any systematic evaluation using the same sample sets across different complexity levels, and using optimized, highly parallel conditions to ensure that each workflow is at the apex of their performance scale. Our study provides a rigorous, data-driven benchmark with respect to the differences between the two cross-linkers.

- "For interactome mapping of mammalian cells, neither suffices but DSSO cross-linking combined with fractionation produces better results."
Is this search engine dependent or independent? Please show data of 5 independent search strategies.
Response 8: We appreciate the reviewer's concern regarding search-engine dependency. To address this, we would like to refer to our Nature Communications (Chen et al.) paper in 2019, in which we systematically evaluated a series of cross-link search engines, including xiSEARCH and MetaMorpheusXL, among others. Our data demonstrated that only a select few engines (pLink 1, pLink 2, Kojak, MetaMorpheusXL, and Xolik) are computationally capable of executing human proteome-scale searches. More importantly, when applied at this massive scale, the identification sensitivities of Kojak, MetaMorpheusXL, and Xolik plummeted to 56.9%, 2.4%, and 5%, respectively, relative to our baseline. Because these alternative search strategies are not compatible with the mammalian datasets, implementing them into this specific evaluation is difficult. Even if we managed to get them to finish the search, they would introduce massive computational variations, which would mask the actual chemical differences between DSS and DSSO in the liquid phase and the gas phase.

a

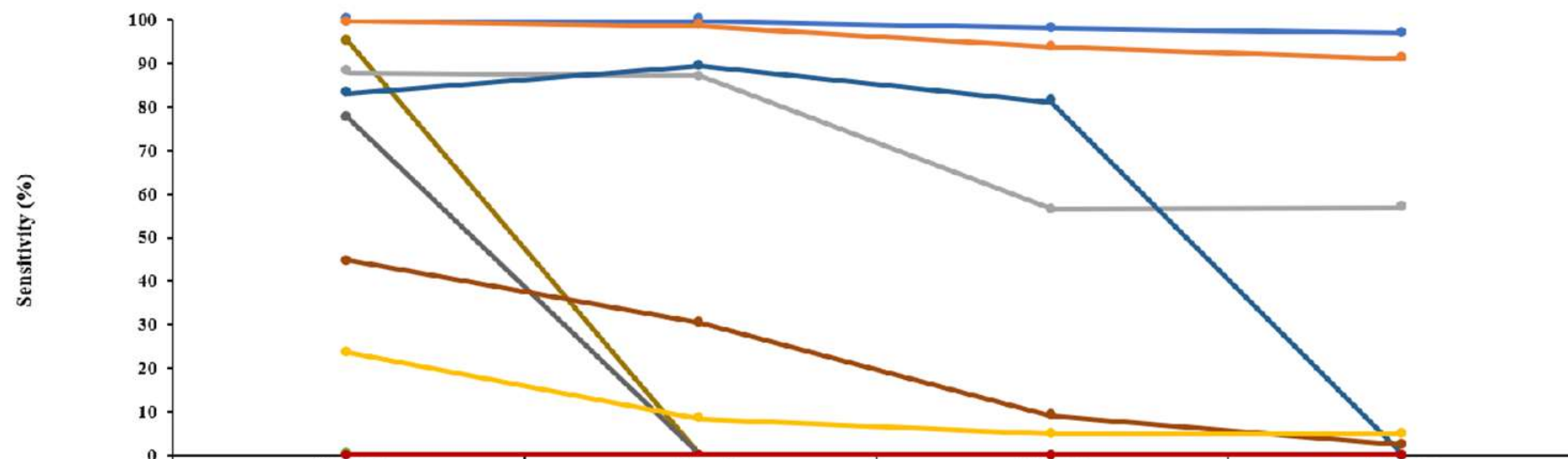

	Original	+ E. coli	+ Worm	+ Human
pLink 2	99.8	99.8	98.0	97.0
pLink 1	99.6	98.5	93.7	91.0
StavroX	94.9	0.0	0.0	0.0
Kojak	87.9	87.1	56.5	56.9
PP	83.1	89.5	81.1	0.0
Xi	77.4	0.0	0.0	0.0
MetaMorpheusXL	44.8	30.3	9.0	2.4
Xolik	23.6	8.3	5.0	5.0
Xilmass	0.1	0.0	0.0	0.0
xQuest	0.0	0.0	0.0	0.0

b

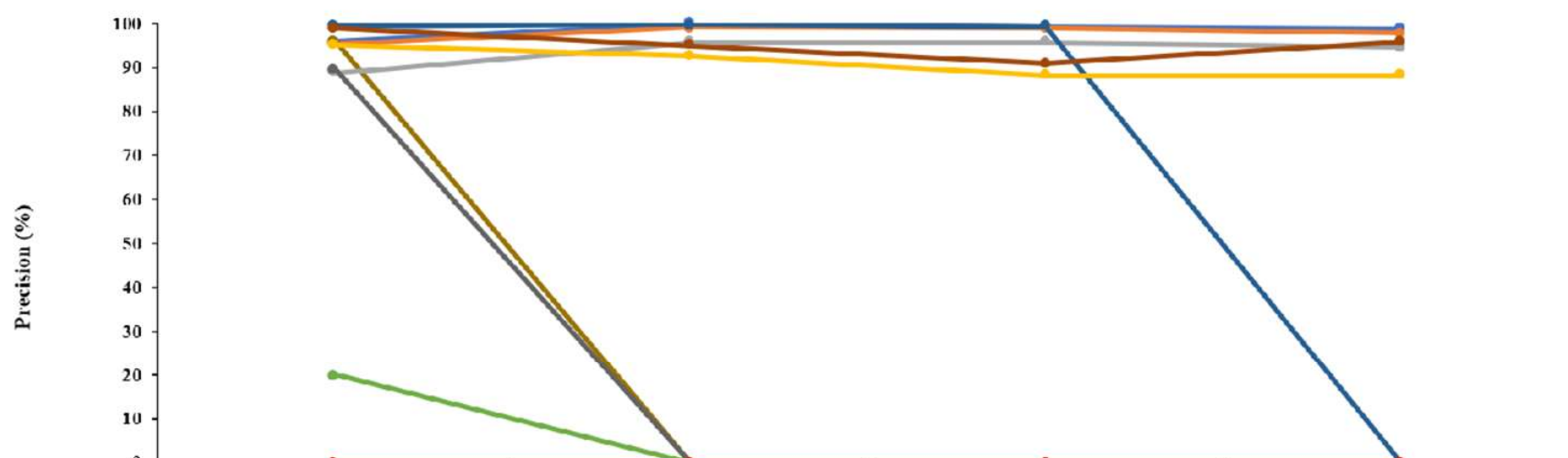

	Original	+ E. coli	+ Worm	+ Human
pLink 2	95.9	99.9	99.1	98.7
pLink 1	95.3	99.1	98.8	97.9
StavroX	95.9	0.0	0.0	0.0
Kojak	88.7	95.5	95.5	94.7
PP	99.4	99.8	99.2	0.0
Xi	89.7	0.0	0.0	0.0
MetaMorpheusXL	98.9	94.8	91.0	95.7
Xolik	94.9	92.6	88.2	88.2
Xilmass	20.0	0.0	0.0	0.0
xQuest	0.0	0.0	0.0	0.0

Supplementary Figure 19. Performance evaluation on the Synthetic-BS3 dataset. The **a)** sensitivity and **b)** precision of ten search engines when searching against original database plus entrapment databases of different sizes. Search engines were sorted by sensitivity in original database in descending order.

- „warheads" - A very strange expression that I had never heard before and would avoid using in a professional context

Response 9: We respectfully disagree with the assessment that "warhead" is unprofessional. This term is standard nomenclature in chemical biology and chemoproteomics to describe the reactive functional groups of chemical probes that target specific amino acid residues. Its usage is well-documented in the literature, for example:

Nature Chemical Biology (2005): "...as this 'warhead' targets diverse families of cysteine proteases" (DOI: 10.1038/nchembio728).
Nature (2016): "...with explicit chloroacetamide and acrylamide warheads" (DOI: 10.1038/nature18002).
Nature Communications (2021): "...which lacks the diazirine-alkyne crosslinking warhead" (DOI: 10.1038/s41467-021-25973-x).

If I have misunderstood something in the manuscript or missed something, I would like to apologize to the authors in advance.

Reviewer #2:

This manuscript provides a timely and systematic evaluation of the trade-offs between non-cleavable (i.e., DSS) and cleavable (i.e., DSSO) cross-linkers. Building significantly on the work by Kolbowski et al. (Ref. 31 of the manuscript), the authors move beyond the "how" of fragmentation to provide evidence-based guidelines on "when" to use specific reagents based on sample complexity. A "crossing point" for the two cross-linkers appears to be between a bacterial-level lysate (thousands of proteins) and a mammalian-level lysate (over ten thousand proteins). The identification of the "spacer arm" volume (71.9% greater for DSS) as a primary driver for identification count in low-complexity samples is also a notable contribution. I have several critiques:

1. The authors attribute the performance gap primarily to the longer and more flexible spacer arm of DSS. To verify that this is not a result of inherent differences in chemical reactivity, the authors should provide a comparison of mono-link identifications for both reagents. This would clarify if the "warhead" efficiency is truly equivalent before concluding that the 3D "sampling volume" is the differentiator.

Response 10: Thank you for this insightful suggestion. To address this, we have extracted and compared the mono-link identifications for both reagents (data provided below).

Across the dataset of 8 purified proteins or protein complexes, that of yeast ribosome, *E. coli* lysate, or HEK293T lysate, the number of DSS mono-link PSMs differed from that of DSSO by -13%, 14%, -1.3%, or 17%, respectively. At the peptide level, these differences were 16%, 20%, -4%, or 15%, respectively. As such, we have no compelling evidence to conclude definitely that the NHS ester group of DSS is more reactive than the NHS ester group of DSSO.

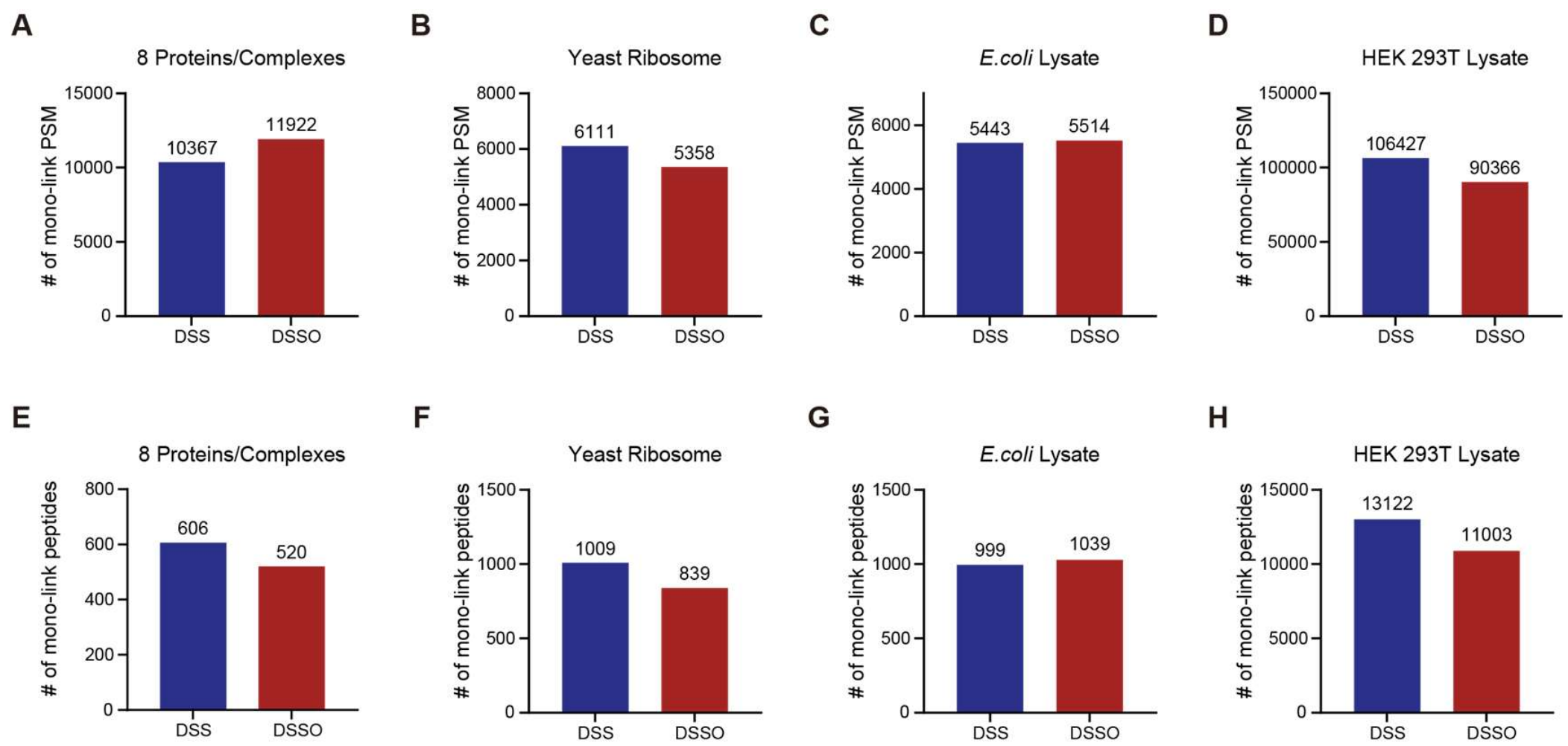

2. In the mammalian lysate experiments (Figure 1G), the authors switch from DSS to its sulfonated analog, BS3, to overcome solubility issues. While the cross-linked products are identical, the initial reactivity and partitioning in a complex lysate may differ between the hydrophobic DSS and the hydrophilic BS3. The authors should discuss or experimentally address how this switch might impact the validity of the comparison with DSSO. It would be even better to perform a similar comparison between BS3 and DSS (see my last comment).

Response 11: Thanks for raising this important point regarding the potential differences in reactivity and partitioning between DSS and BS3. In a previous publication from our laboratory (Ding et al., *Anal Chem*, 2016), we compared DSS and BS3 on BSA, Aldolase and the CNGP complex; in that study, the hydrophobic DSS outperformed the hydrophilic BS3 (yielding 159 vs. 126 identified peptide pairs, respectively).

To explicitly address your concern, we conducted additional side-by-side comparisons of DSS and BS3 on the CD36-ApoD complex and the yeast ribosome (with two biological replicates for each sample). In these new experiments, DSS performed similarly to or slightly better than BS3 (51 vs. 50 peptide pairs s on the CD36-ApoD complex, and 880 vs. 870 cross-links on the yeast ribosome). Taken together, these data indicate that while DSS may have a slight advantage in certain contexts, BS3 exhibits highly comparable cross-linking efficiency. Therefore, utilizing BS3 to circumvent solubility issues in the mammalian lysate should not significantly compromise the validity of our comparison with DSSO.

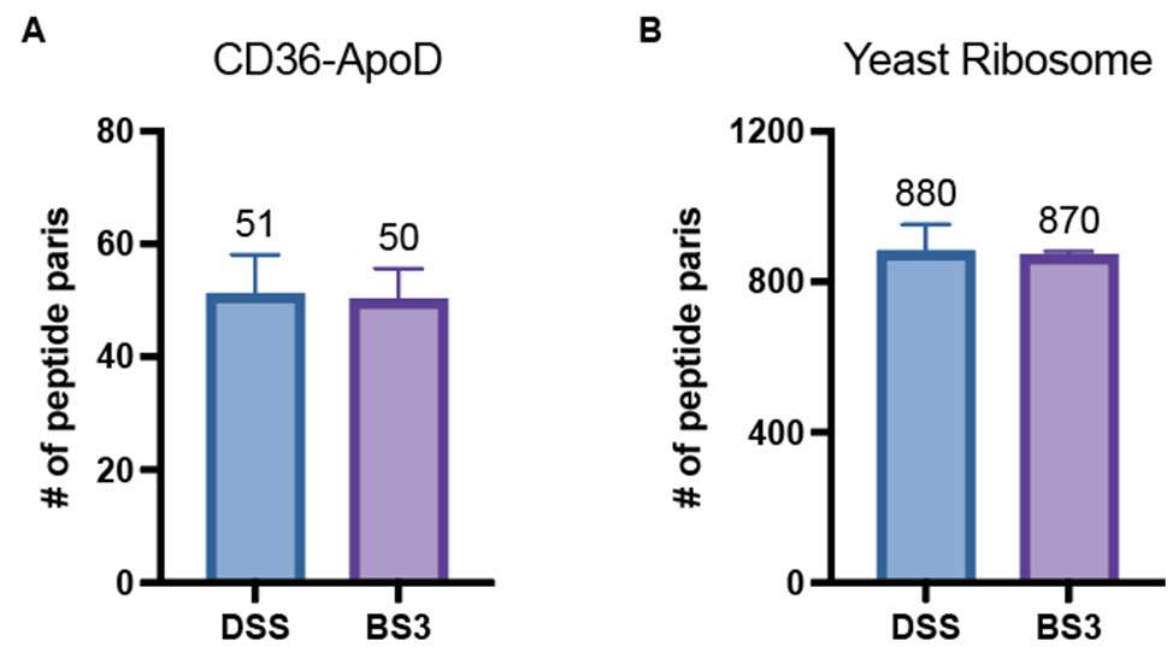

3. The "85% fragment ion coverage rule" is presented as a requirement for proteome-somewhat coarse. Could the authors provide more granular data or a clearer statistical justification for why 85%-rather than 75% or 80%-is the critical threshold?

Response 12: We thank the reviewer for this insightful comment and the opportunity to clarify our threshold selection. To provide a more granular statistical justification, we subdivided the fragment ion coverage data into five narrower bins: (0%, 60%], (60%, 70%], (70%, 80%], (80%, 90%], (90%, 100%].

When searching against database containing not only the E. coli proteins but also a varying number of human proteins, we observed performance drop-off at lower coverages. Specifically, spectra falling into the (70%, 80%] bracket and below could not maintain our target high sensitivity (>90%). Because robust, high sensitivity (>90%) is only preserved within the (80%, 90%] bracket and above, we selected 85%—the midpoint of this critical boundary bracket—as a conservative and reliable threshold to ensure optimal search performance for proteome-wide applications.

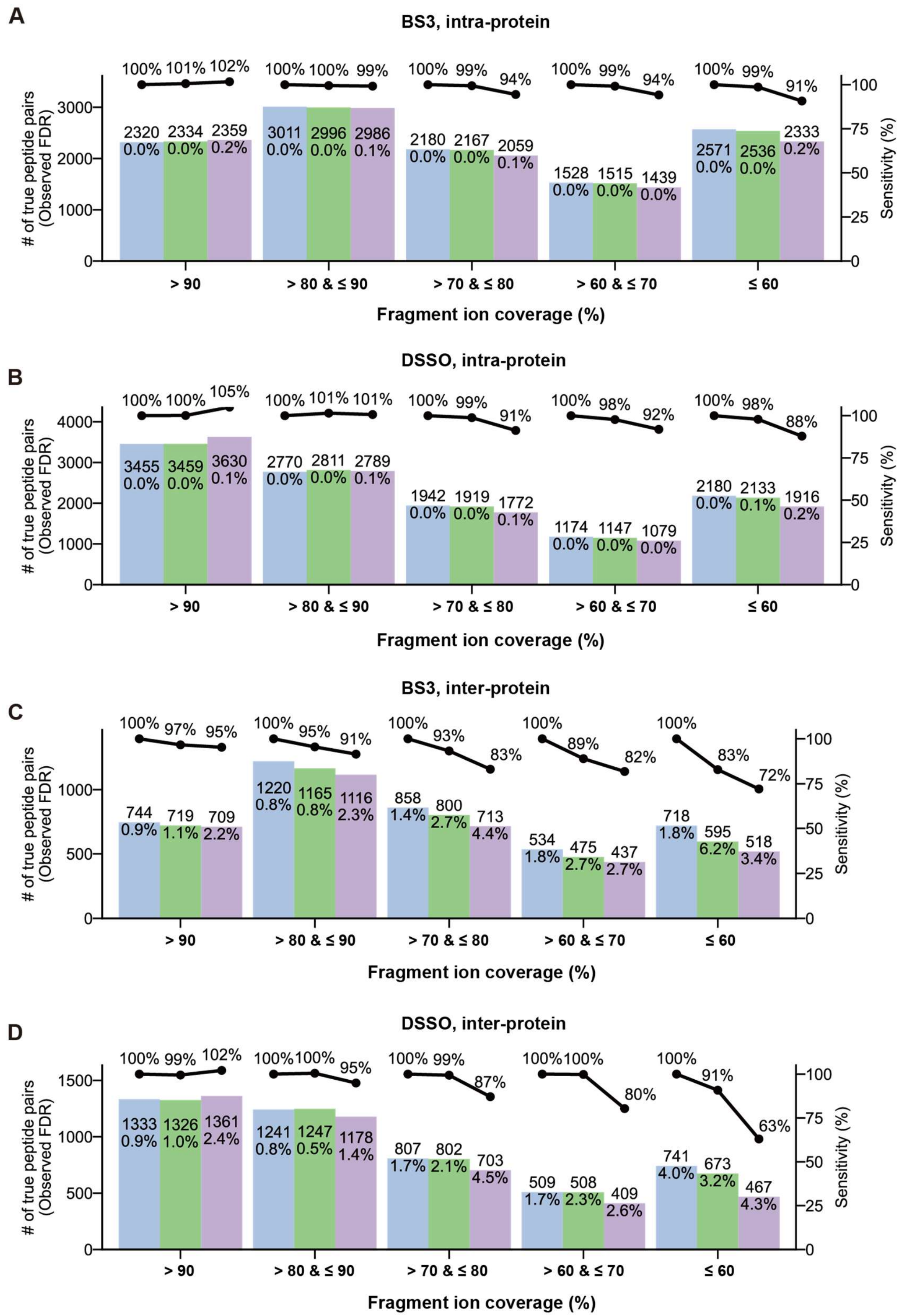

Additionally, the presentation of Figure 5 requires clarification, specifically:

> The definition of "Observed FDR" in the context of the re-identification plots (Panels C-F) should be explicitly stated in the legend.

Response 13: The data used in Fig. 5C–F originated from a previously published study (Lenz, S. et al., 2021, Nat. Commun.). In that study, a benchmarked *E. coli* dataset was

constructed using a fractionation method to evaluate search engine precision (actual FDR). Specifically, intra-fraction protein–protein interactions (PPIs) were defined as true positives, whereas inter-fraction cross-links were defined as false positives. We adopted this identical methodology and utilized "Supplementary Data 7: All plausible PPIs based on SEC fractionation" provided by the original paper to determine true positive and false positive PPIs. In our work, since we added human protein databases of varying sizes as entrapment, any identified peptide pair in which one or both peptides matched to a human protein sequence was also considered a false positive. The observed FDR was calculated as the proportion of false positive results among all identifications.

> The method for calculating Precision and Sensitivity in the simulated datasets should be more clearly linked to the "true" matches in the simulation.

Response 14：Details regarding the generation of simulated spectra are provided in the Methods section (Page 17). A total of 10,000 simulated cross-linked spectra (denoted as x) were evaluated. The identities of the cross-linked peptide pairs for these simulated spectra were known by design: each peptide pair was created by randomly selecting two peptides from the 100-protein database and the mass values of theoretical fragment ions were calculated for each artificially generated cross-link. A true positive match was defined as an instance where the final identification result given by the search engine matched exactly to the source peptide pair used to generate the corresponding simulated spectrum. After applying false discovery rate (FDR) control, the total number of matches reported by the search engine was denoted as y, and the subset of true positive matches within y was denoted as z. Precision was then calculated as z/y, and sensitivity was calculated as z/x.

Reviewer #3:

Cao et al. revisit the topic of cleavability of chemical cross-linking reagents for structural mass spectrometry/proteomics applications, comparing the performance of the non-cleavable DSS and the cleavable DSSO in the same computational framework (pLink 3). The authors find that DSS consistently outperforms DSSO for samples of lower complexity (ranging from individual proteins up to E. coli) while DSSO performs better at the human proteome scale. The former effect is, for the first time, attributed to the increased span of the linker arm as assessed by MD simulations: Although the difference in spacer length between the two reagents appears small (approx. 1.3 Å), this results in a disproportionally higher accessible volume for DSS. At the same time, the non-cleavable DSS shows less efficient backbone cleavage, as previously also observed by others, which appears to be the most relevant effect for DSSO's improved identification rates in large search spaces.

The manuscript challenges the default assumption of some researchers in the cross-linking community that cleavable reagents are always superior to non-cleavable ones of a similar type. As a side note, it also confirms my personal experience for low-

complexity samples. Nevertheless, I was surprised by the magnitude of this effect for individual proteins (Figure 2) in a side-by-side comparison with different reagents with a slightly shorter linker span.

In summary, I find this work very interesting and relevant for the cross-linking community and those researchers who use cross-linking as an auxiliary technique in their structural or systems biology projects. I have mostly minor comments for clarifications and corrections that are summarized below.

Comments:

Page 6, line 12: replications > replicate
Response 15: We thank the reviewer for his/her encouraging remarks and for pointing out the mistakes in the manuscript. The word "replications" is replaced by "replicate" on Page 5, line 12.

Page 8, figure 1: In panel C, the x-axis labeling MW(Da) = 78 KD etc. needs to be corrected. For example, write MW(kDa) and then just give numbers. Make sure to distinguish between cross-links (the products) and cross-linking (the process) in the figure legend.
Response 16: Thanks for your advice. It has been corrected.

Page 16: "this study joins an early one ...". Please add a reference here.
Response 17: We apologize for this omission. The appropriate reference [31, Kolbowski et al., *Anal Chem*, 2022] is added to this sentence on Page 10 line 9.

Page 16: Here, the authors refer to synthetic peptides generated for this study (as opposed to re-searching a dataset of synthetic peptides from another lab, ref. 37). No further information about this is given except in Supplementary Table 1. More information needs to be added to the method section, and it should be clear throughout the manuscript (main and SI figures) which synthetic dataset is used where.
Response 18: We have a manuscript in preparation detailing the synthesis method. Briefly, synthesis of cross-linked peptide pairs was initiated by swelling Boc-Pep1-CTC resin in DMF. The resin-bound peptide was subsequently treated with a cross-linker solution of either DSS or DSSO, in DMF containing triethylamine (2%) as a base. Completion of the cross-linking reaction was confirmed via a ninhydrin test. Following extensive washing with DMF to remove unreacted cross-linker and base, the second peptide, Boc-Pep2-OH, was coupled to the resin-bound intermediate under similar basic conditions. The resulting cross-linked peptide-resin was sequentially washed with DMF and methanol and then dried under vacuum. Finally, the target peptide was simultaneously cleaved from the solid support and globally deprotected using a cleavage reagent composed of trifluoroacetic acid, tri-isopropylsilane, and water (95:2.5:2.5). The crude product was isolated from the filtrate by precipitation with cold

diethyl ether, followed by centrifugation, further washing, and vacuum drying to yield the final compound for mass spectrometric analysis. The synthesis method is described in detail in a separate manuscript under preparation, in which the data of the synthetic peptides take a central position in supporting the software development of pLink 3. Yes, users across the globe have embraced pLink 3, but the pLink 3 manuscript is still in preparation 1+ year after the software release.

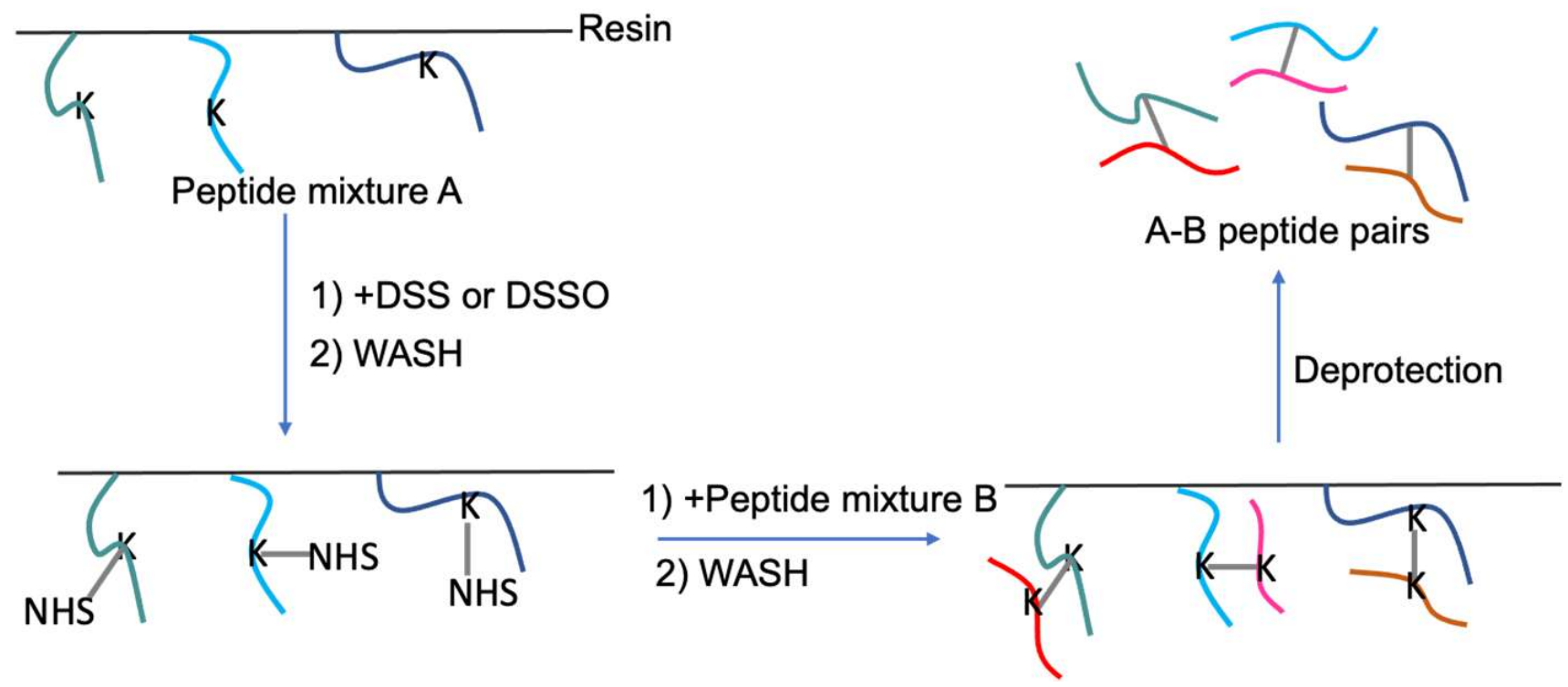

We have revised the figure legends for all Supplementary Figures, including Supplementary Figures 2 and 3, to explicitly state the corresponding sample and source data.

Page 19: "with extensive fractionation (refs)" Please add the references here.
Response 19: We thank the reviewer for catching this oversight. The placeholder has been removed, and the appropriate references have now been added to this sentence in the revised manuscript (Page 12 line 20).

Page 21: Please give the reaction time for the HEK293 cross-linking experiment.
Response 20: The reaction time and temperature are added to the Materials and Methods section. “HEK293T cells were lysed in PBS buffer, 1mL (1mg/mL) lysate was treated with 1 mM of DSSO or BS3 for 45 min at room temperature.”

Page 22: The specific versions of pLink 3 (and Scout) should be stated here. Is the version with the doublet functionality publicly available or was this feature added for in-house use only?
Response 21: Certainly. The specific versions of pLink 3 and Scout are now indicated in the Materials and Methods section of the revised manuscript. Further, we confirm that the version featuring the doublet functionality is publicly available, not restricted to in-house use. Both the pLink 3-PepIon and pLink 3-Doublet versions have been uploaded to GitHub, and this link is now provided in the Code Availability section: https://github.com/daheitu/filtering-enabled-version-of-pLink.

Supplementary Figure 3: Do the authors have any explanation about the large differences in CSMs between DSSO and DSBU for Scout? Did you observe any major differences in spectral properties of the identifications for your own software, pLink (for examples, in some parameters that pLink uses for scoring)?

Response 22: Good question. While the specific algorithmic design and internal scoring heuristics of Scout are not entirely accessible to us, our empirical analysis of the spectra provides a likely explanation. We observed that the signature mass doublets generated by the cleavage of DSBU are detected at a noticeably lower frequency in the MS/MS spectra compared to those from DSSO (based on synthetic peptides dataset, PXD014337; see attached figure below). Because Scout operates as a doublet-dependent search engine, this reduction in detectable signature doublets inherently decreases its identification sensitivity, leading to the large difference in CSMs.

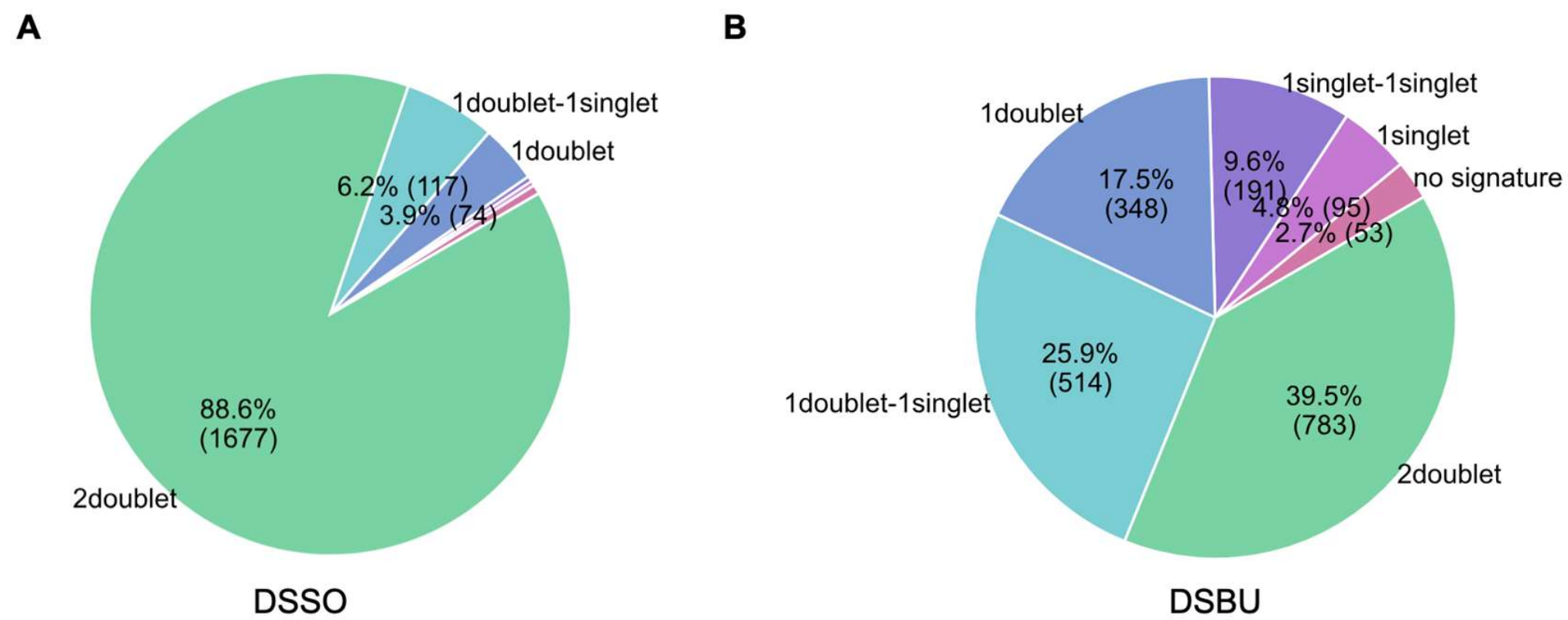

Supplementary Figure 4: I assume that the values given on the x-axis are in Å but this should be explicitly mentioned.

Response 23: Sorry about it. We have updated the x-axis label in Supplementary Figure 4 to explicitly include the unit "Å" (Angstroms).

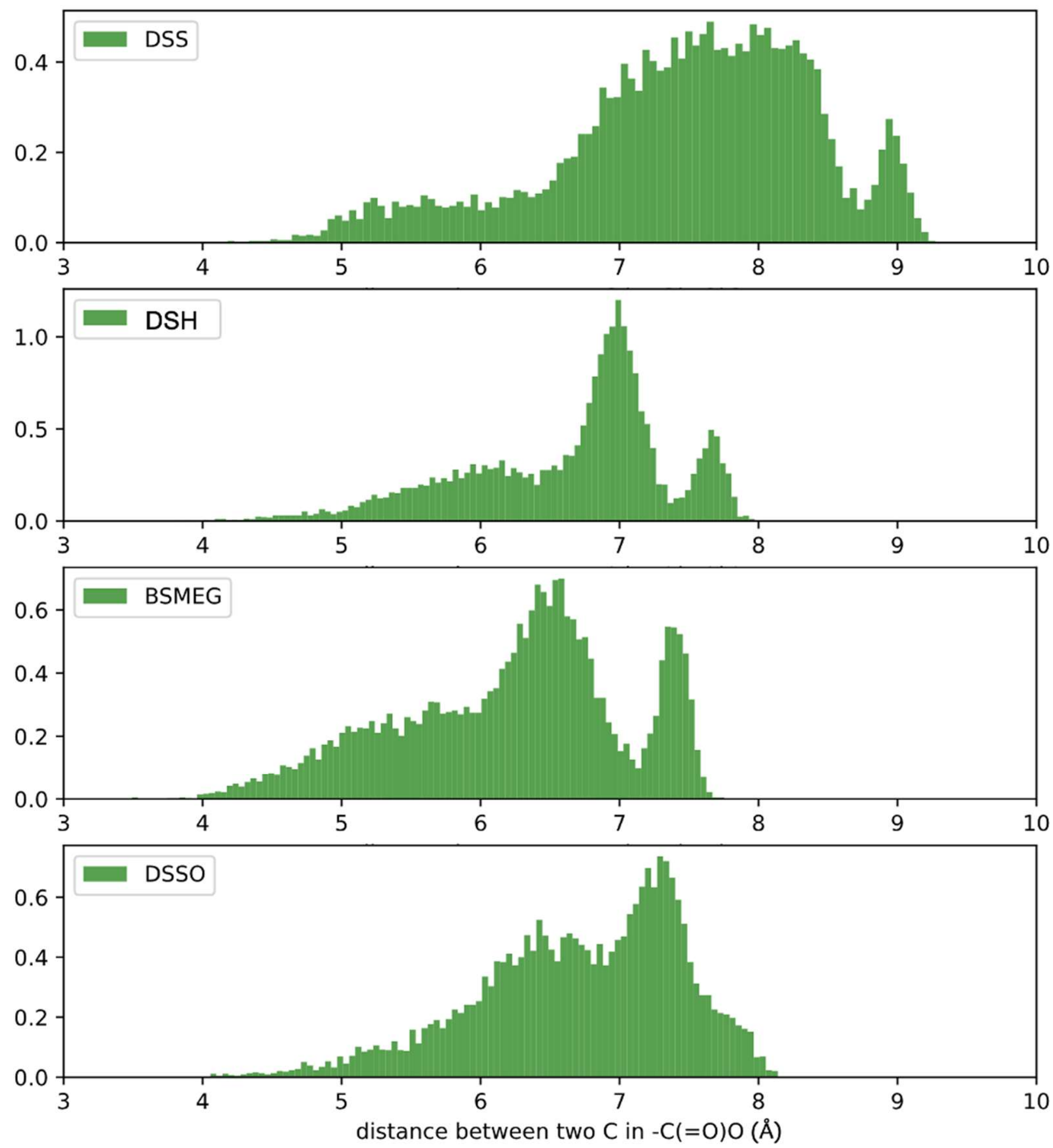

Supplementary Figure 5: The figure labels say DSSC, is this the same as DSH?

Response 24: We thank the reviewer for catching this inconsistency. Yes, "DSSC" was our previous internal designation for the DSH cross-linker. We have now corrected the labels in Supplementary Figure 5 to read "DSH" to match the rest of the manuscript.

A

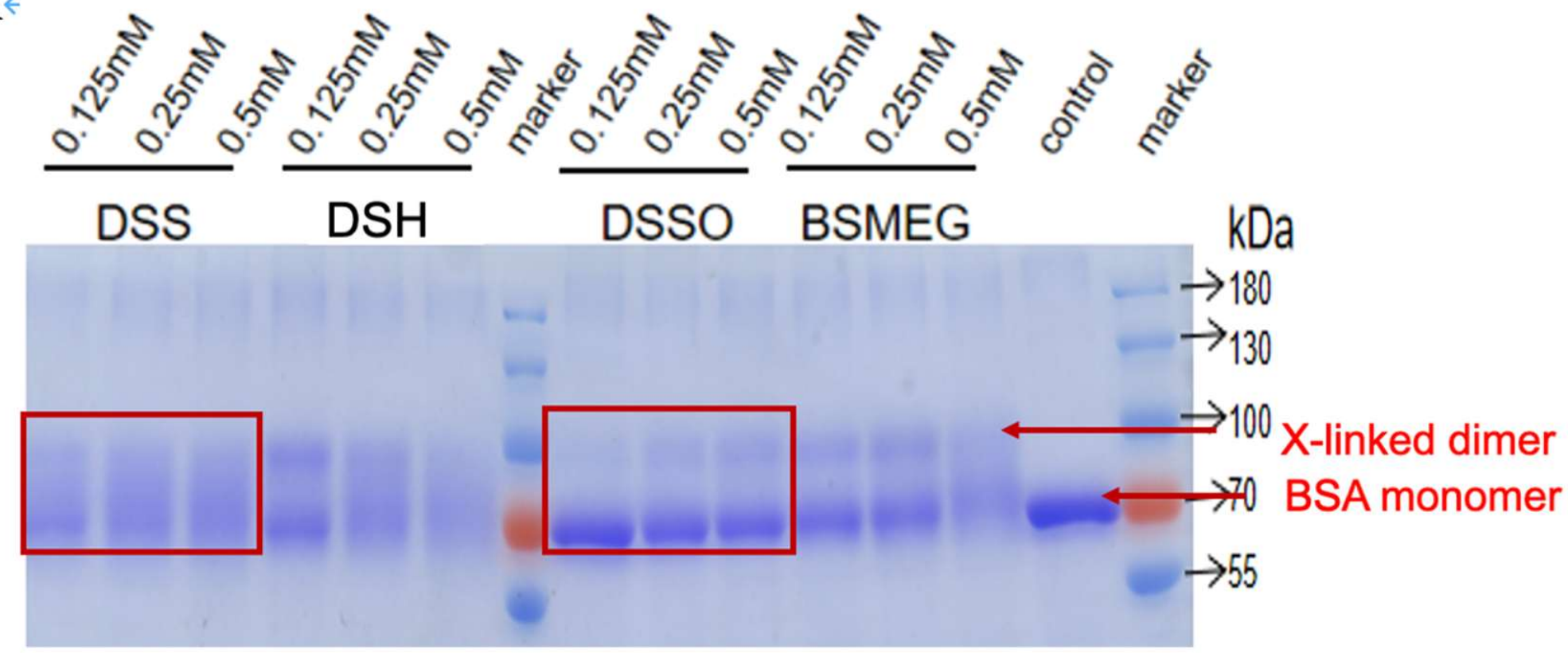

B

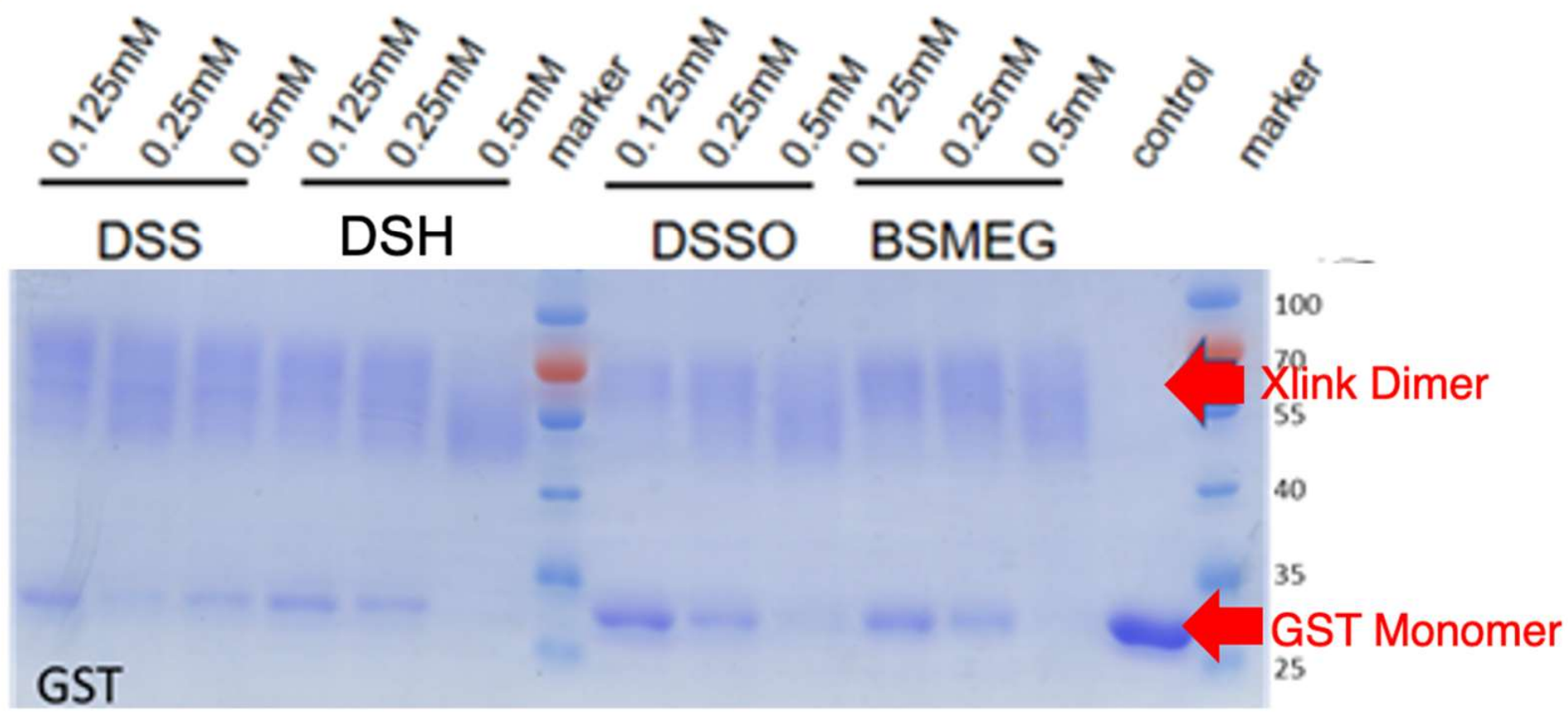

Supplementary Figures, general: Please always make clear which samples correspond to the results shown, e.g. SF Figure 2 and 3.

Response 25: Thanks for the helpful suggestion. We have revised the figure legends for all Supplementary Figures, including Supplementary Figures 2 and 3, to explicitly state the corresponding sample and data source information.

Response to editorial requests

On a more editorial level, we would ask you to address the following issues:

- Please provide a .docx formatted version of the manuscript text (including legends for main figures, EV figures and tables). Please make sure that the changes are highlighted to be clearly visible.

Response: We have provided a .docx formatted version of the revised manuscript. As requested, this file includes all figure and table legends, and all changes have been clearly highlighted.

- Please provide individual production quality figure files as .eps, .tif, .jpg (one file per figure). When assembling figures, please refer to our figure preparation guideline in order to ensure proper formatting and readability in print as well as on screen:
https://media.springernature.com/original/springer-cms/rest/v1/content/27825798/data/v1

Response: In accordance with the journal's guidelines, we have uploaded individual, production-quality .tif files for each figure.

-Please provide a .docx formatted letter INCLUDING the reviewers' reports and your detailed point-by-point responses to their comments. As part of the EMBO Press transparent editorial process, the point-by-point response is part of the Review Process File (RPF), which will be published alongside your paper.

Response: We have provided a detailed, point-by-point response letter in .docx format. As requested, this file includes the complete reviewer reports alongside our specific replies to each comment.

-Please note that all corresponding authors are required to supply an ORCID ID for their name upon submission of a revised manuscript.

Response: We have provided the ORCID IDs for all corresponding authors.

M.-Q. D. (E-mail: *dongmengqiu@nibs.ac.cn*) https://orcid.org/0000-0002-6094-1182
S.-M. H. (E-mail: *smhe@ict.ac.cn*) https://orcid.org/0000-0002-2434-7194
X.-B. Q. (E-mail: *qixiangbing@nibs.ac.cn*) https://orcid.org/0000-0002-7139-5164

-We replaced Supplementary Information with Expanded View (EV) Figures and Tables that are collapsible/expandable online. A maximum of 5 EV Figures can be typeset. EV Figures should be cited as 'Figure EV1, Figure EV2" etc... in the text and their respective legends should be included in the main text after the legends of regular figures.

Additional Tables/Datasets should be labeled and referred to as Table EV1, Dataset EV1, etc. Legends have to be provided in a separate tab in case of .xls files.

Alternatively, the legend can be supplied as a separate text file (README) and zipped together with the Table/Dataset file.

For the figures and tables that you do NOT wish to display as Expanded View figures, they should be bundled together with their legends in a single PDF file called *Appendix*, which should start with a short Table of Content. Each legend should be below the corresponding Figure/Table in the Appendix. Appendix figures and tables should be referred to in the main text as: "Appendix Figure S1, Appendix Figure S2, Appendix Table S1" etc. See detailed instructions regarding expanded view here: https://link.springer.com/journal/44320/submission-guidelines#cms-Expanded-View-data.
Response: We have opted to place all supplementary figures and tables into a single Appendix PDF file. In accordance with your guidelines, we have included a short Table of Contents at the beginning of the file, placed the legends directly below their corresponding figures/tables, and updated all in-text citations to the required nomenclature (e.g., 'Appendix Figure S1', 'Appendix Table S1').

-Before submitting your revision, primary datasets (and computer code, where appropriate) produced in this study need to be deposited in an appropriate public database.
Please remember to provide a reviewer password if the datasets are not yet public.
The accession numbers and database should be listed in a formal "Data Availability" section (placed after Materials & Method) that follows the model below (see also https://link.springer.com/partners/embo-press/editorial-policies#Data%20availability%20statement). Please note that the Data Availability Section is restricted to new primary data that are part of this study.
Data availability
The datasets (and computer code) produced in this study are available in the following databases:
- RNA-Seq data: Gene Expression Omnibus GSE46843 (https://www.ncbi.nlm.nih.gov/geo/query/acc.cgi?acc=GSE46843)
- [data type]: [name of the resource] [accession number/identifier/doi] ([URL or identifiers.org/DATABASE:ACCESSION])
*** Note - All links should resolve to a page where the data can be accessed. ***
Response: In accordance with the journal's policy, we have uploaded our primary mass spectrometry data to ProteomeXchange and our code to GitHub. All accession numbers, resolvable links, and access details are now listed in a dedicated 'Data and Code Availability' section using the requested format.

- At EMBO Press we ask authors to provide source data for the main manuscript figures. You will receive a separate email with instructions for providing source data with your revised manuscript, including how to upload and organize the files.
Additional information on source data and instruction on how to label the files are available < https://link.springer.com/journal/44320/submission-guidelines#cms-

Source-data >
Response: We have provided the source data for the main manuscript figures as request.

- Our journal encourages inclusion of *data citations in the reference list* to directly cite datasets that were re-used and obtained from public databases. Data citations in the article text are distinct from normal bibliographical citations and should directly link to the database records from which the data can be accessed. In the main text, data citations are formatted as follows: "Data ref: Smith et al, 2001". In the Reference list, data citations must be labeled with "[DATASET]". A data reference must provide the database name, accession number/identifiers and a resolvable link to the landing page from which the data can be accessed at the end of the reference. Further instructions are available at < https://link.springer.com/journal/44320/submission-guidelines#cms-Reference-guidelines >.
Response: We have provided the accession numbers for all public datasets in the respective figure legends, and the original papers associated with these datasets have been appropriately cited.

- We updated our journal's competing interests policy in January 2022 and request authors to consider both actual and perceived competing interests. Please review the policy (https://link.springer.com/partners/embo-press/editorial-policies#Competing%20interest%20disclosures) and update your competing interests if necessary.
Please use the heading "Disclosure statement and competing interests".
Response: We have reviewed the updated policy and added the 'Disclosure statement and competing interests' section to the manuscript, as requested.

- All Materials and Methods need to be described in the main text using our 'Structured Methods' format. According to this format, the Methods section includes a Reagents and Tools Table (listing key reagents, experimental models, software and relevant equipment and including their sources and relevant identifiers) followed by a Methods and Protocols section describing the methods, ideally using a step-by-step protocol format. The aim is to facilitate adoption of the methodologies across labs.

Please download and fill our Reagents and Tools Table template (.docx), which you can find in our author guidelines: https://link.springer.com/journal/44320/submission-guidelines#structuredmethods.

When submitting your revised manuscript, please do not include the Reagents and Tools Table in the Methods section of the manuscript but upload it as a separate file choosing the file type "Reagent Table".
Response: The Reagents and Tools Table has been filled out using the official template and uploaded as a separate file designated as a ‘Reagent Table’.

-Regarding data quantification:

Please ensure to specify the name of the statistical test used to generate error bars and P values, the number (n) of independent experiments (please specify technical or biological replicates) underlying each data point and the test used to calculate p-values in each figure legend. Discussion of statistical methodology can be reported in the materials and methods section, but figure legends should contain a basic description of n, P and the test applied.
Graphs must include a description of the bars and the error bars (s.d., s.e.m.).
Please also include scale bars in all microscopy images.
Response: We have updated the figure legends to explicitly state the exact P values, the sample sizes (n, specifying biological replicates), and the definitions of the error bars.

- Please provide a "standfirst text" summarizing the study in one or two sentences (approximately 250 characters, including space), three to four "bullet points" highlighting the main findings and a "visual abstract" (550px width and 400-600 px height, PNG format) to highlight the paper on our homepage.
Please refer to published papers for examples.
Response: "standfirst text" and "visual abstract" are attached below.
Standfirst text: Through a thorough experimental comparis
on, this study finds that a non-cleavable cross-linker DSS yields more identifications of cross-linked peptide pairs in less complex samples such as purified protein complexes and E. coli lysate, whereas a cleavable cross-linkers DSSO performs better on mammalian whole-cell lysate.
Visual abstract：

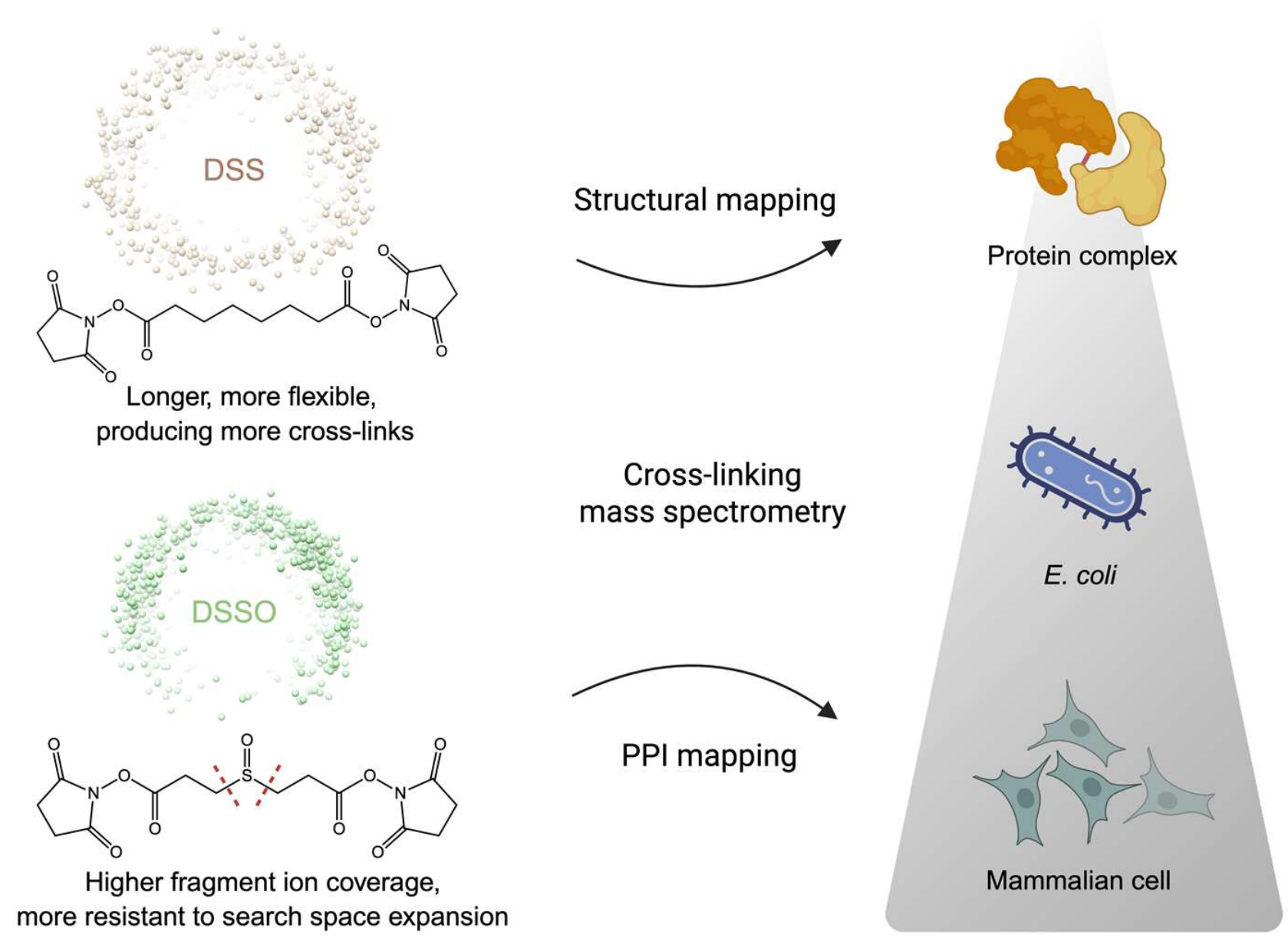

21st May 2026

Manuscript Number: MSB-2026-13676R
Title: To cleave or not to cleave: a systemic evaluation of DSS versus DSSO for cross-linking mass spectrometry analysis
Author: Yong Cao
Peng-Zhi Mao
Qing-Cui Wu
Zhen-Lin Chen
Jin-Cai Yang
Yue Zhao
Adalet Memetimin
Hao Chi
Xiangbing Qi
Si-Min He
Mengqiu Dong

Dear Mengqiu,

Thank you for the submission of your revised manuscript to Molecular Systems Biology. We have now received the enclosed report from the reviewer who was asked to re-assess it. As you will see, the reviewer is now supportive, and I am pleased to inform you that we will be able to accept your manuscript pending the following editorial-level amendments:

1. Please add the section heading "Abstract" before the abstract.

2. Please remove the "Author contribution" section from the manuscript file.

3. The references need to be formatted according to the Molecular Systems Biology reference style. Please list up to 10 co-authors of a paper before adding et al. in the reference list. Citations should be listed in alphabetical order.

4. Please add missing callouts for specific panels of Appendix figures.

5. In the Appendix file, "Supplementary Information" should be replaced with "Appendix for [manuscript title]" (the author and affiliation list is not necessary). Highlights should also be removed from the text, as the Appendix PDF will not be typeset.

6. The following funding information is included in the manuscript file but is missing from the online submission system and therefore also needs to be added there: Beijing Natural Science Foundation (No. Z230020); the Beijing Municipal Government; and Tsinghua University.

7. Author checklist: all three corresponding authors need to be added.

8. "Data and Code Availability" should be renamed to "Data Availability".

9. "Disclosure statement and competing interests" should be renamed to "Disclosure and competing interests statement".

10. Please provide a "standfirst" summarizing the study in one or two sentences (approximately 250 characters, including spaces), as well as three to four bullet points highlighting the main findings, in a DOCX file.

Please also provide a visual abstract (550 px wide × 300 px high) in a separate PNG file for highlighting the paper on our homepage.

11. Source data for main figures should be uploaded as one (zipped) file /figure, and named as "manuscriptID_SourceDataForFigure x".

12. Please address the following issue in the figure legends:
- Please note that the exact p values are not provided in the legends of 5B.
- Please indicate the statistical test used for data analysis in the legends of figure 5B.
- Please note that the box plots need to be defined in terms ofminima, maxima, centre, bounds of box and whiskers, and percentile in the legends of figure 5B.

13. Sections need to be named and the order should be corrected to: Title page - Abstract - Introduction - Results - Discussion - Methods - Data Availability - Acknowledgements - Disclosure and Competing Interests Statement - References - Figure Legends

- Table(s) - Expanded View Figure Legends.

I look forward to receiving the revised manuscript soon.

Kind regards,
Jingyi

Jingyi Hou, PhD
Senior Editor
Molecular Systems Biology

If you do choose to resubmit, please click on the link below to submit the revision online before 20th Jun 2026.

https://msb.msubmit.net/cgi-bin/main.plex

Reviewer #2:

The authors have addressed all my major and minor concerns by providing comprehensive experimental data, relevant references, and detailed theoretical explanations. They have also updated the methods and figure captions in the main text. Though the results focus primarily on the comparison between DSS/BS3 and DSSO/DSBU, they provide interesting perspectives that contribute to the field of cross-linking technology. I therefore recommend acceptance of the manuscript for publication.

All editorial and formatting issues were resolved by the authors.

27th May 2026
Manuscript number: MSB-2026-13676RR
Title: To cleave or not to cleave: a systemic evaluation of DSS versus DSSO for cross-linking mass spectrometry analysis

Dear Dr Dong,

Thank you again for sending us your revised manuscript. We are now satisfied with the modifications made and I am pleased to inform you that your paper has been accepted for publication.

Your manuscript will be processed for publication by EMBO Press. It will be copy edited and you will receive page proofs prior to publication. Please note that you will be contacted by Springer Nature Author Services to complete licensing and payment information.

You may qualify for financial assistance for your publication charges - either via a Springer Nature fully open access agreement or an EMBO initiative. Check your eligibility: https://link.springer.com/journal/44320/how-to-publish-with-us

Should you be planning a Press Release on your article, please notify the production team via this form in order to coordinate publication and release dates: https://forms.cloud.microsoft/e/eH4A8JVP6g

If you have any questions, please do not hesitate to contact the Editorial Office. Thank you for your contribution to Molecular Systems Biology.

Sincerely,
Jingyi

Jingyi Hou, PhD
Senior Editor
Molecular Systems Biology

>>> Please note that it is Molecular Systems Biology policy for the transcript of the editorial process (containing referee reports and your response letter) to be published as an online supplement to each paper. If you do NOT want this, you will need to inform the Editorial Office via email immediately. More information is available here: https://link.springer.com/partners/embo-press/editorial-policies#Peer%20review

EMBO Press Author Checklist

Corresponding Author Name: Meng-Qiu Dong, Si-Min He, Xiang-Bing Qi	
Journal Submitted to: Molecular Systems Biology	
Manuscript Number: MSB-2026-13676	

USEFUL LINKS FOR COMPLETING THIS FORM
The EMBO Journal - Author Guidelines
EMBO Reports - Author Guidelines
Molecular Systems Biology - Author Guidelines
EMBO Molecular Medicine - Author Guidelines

Reporting Checklist for Life Science Articles (updated January
This checklist is adapted from Materials Design Analysis Reporting (MDAR) Checklist for Authors. MDAR establishes a minimum set of requirements in transparent reporting in the life sciences (see Statement of Task: 10.31222/osf.io/9sm4x). Please follow the journal's guidelines in preparing your
Please note that a copy of this checklist will be published alongside your article.

Abridged guidelines for figures
1. Data
The data shown in figures should satisfy the following conditions:
- the data were obtained and processed according to the field's best practice and are presented to reflect the results of the experiments in an accurate and unbiased manner.
- ideally, figure panels should include only measurements that are directly comparable to each other and obtained with the same assay.
- plots include clearly labeled error bars for independent experiments and sample sizes. Unless justified, error bars should not be shown for technical
- if n<5, the individual data points from each experiment should be plotted. Any statistical test employed should be justified.
- Source Data should be included to report the data underlying figures according to the guidelines set out in the authorship guidelines on Data

2. Captions
Each figure caption should contain the following information, for each panel where they are relevant:
- a specification of the experimental system investigated (eg cell line, species name).
- the assay(s) and method(s) used to carry out the reported observations and measurements.
- an explicit mention of the biological and chemical entity(ies) that are being measured.
- an explicit mention of the biological and chemical entity(ies) that are altered/varied/perturbed in a controlled manner.
- the exact sample size (n) for each experimental group/condition, given as a number, not a range;
- a description of the sample collection allowing the reader to understand whether the samples represent technical or biological replicates (including how many animals, litters, cultures, etc.).
- a statement of how many times the experiment shown was independently replicated in the laboratory.
- definitions of statistical methods and measures:
  - common tests, such as t-test (please specify whether paired vs. unpaired), simple χ2 tests, Wilcoxon and Mann-Whitney tests, can be unambiguously identified by name only, but more complex techniques should be described in the methods section;
  - are tests one-sided or two-sided?
  - are there adjustments for multiple comparisons?
  - exact statistical test results, e.g., P values = x but not P values < x;
  - definition of 'center values' as median or average;
  - definition of error bars as s.d. or s.e.m.

Please complete ALL of the questions below.
Select "Not Applicable" only when the requested information is not relevant for your study.

Materials

Newly Created Materials	Information included in the manuscript?	In which section is the information available? (Reagents and Tools Table, Materials and Methods, Figures, Data Availability Section)
New materials and reagents need to be available; do any restrictions apply?	Not Applicable	

Antibodies	Information included in the manuscript?	In which section is the information available? (Reagents and Tools Table, Materials and Methods, Figures, Data Availability Section)
For **antibodies** provide the following information:<br>- Commercial antibodies: RRID (if possible) or supplier name, catalogue number and or/clone number<br>- Non-commercial: RRID or citation	Not Applicable	

DNA and RNA sequences	Information included in the manuscript?	In which section is the information available? (Reagents and Tools Table, Materials and Methods, Figures, Data Availability Section)
**Short novel DNA or RNA including primers, probes**: provide the sequences.	Not Applicable	

Cell materials	Information included in the manuscript?	In which section is the information available? (Reagents and Tools Table, Materials and Methods, Figures, Data Availability Section)
**Cell lines:** Provide species information, strain. Provide accession number in repository **OR** supplier name, catalog number, clone number, and/**OR** RRID.	Yes	Materials and Methods
**Primary cultures:** Provide species, strain, sex of origin, genetic modification status.	Not Applicable	
Report if the cell lines were recently **authenticated** (e.g., by STR profiling) and tested for mycoplasma contamination.	Not Applicable	

Experimental animals	Information included in the manuscript?	In which section is the information available? (Reagents and Tools Table, Materials and Methods, Figures, Data Availability Section)
**Laboratory animals or Model organisms:** Provide species, strain, sex, age, genetic modification status. Provide accession number in repository **OR** supplier name, catalog number, clone number, **OR** RRID.	Not Applicable	
**Animal observed in or captured from the field:** Provide species, sex, and age where possible.	Not Applicable	
Please detail **housing and husbandry conditions**.	Not Applicable	

Plants and microbes	Information included in the manuscript?	In which section is the information available? (Reagents and Tools Table, Materials and Methods, Figures, Data Availability Section)
**Plants:** provide species and strain, ecotype and cultivar where relevant, unique accession number if available, and source (including location for collected wild specimens).	Not Applicable	
**Microbes:** provide species and strain, unique accession number if available, and source.	Not Applicable	

Human research participants	Information included in the manuscript?	In which section is the information available? (Reagents and Tools Table, Materials and Methods, Figures, Data Availability Section)
If collected and within the bounds of privacy constraints report on age, sex and gender or ethnicity for all study participants.	Not Applicable	

Core facilities	Information included in the manuscript?	In which section is the information available? (Reagents and Tools Table, Materials and Methods, Figures, Data Availability Section)
If your work benefited from core facilities, was their service mentioned in the acknowledgments section?	Not Applicable	

Design

Study protocol	Information included in the manuscript?	In which section is the information available? (Reagents and Tools Table, Materials and Methods, Figures, Data Availability Section)
If study protocol has been **pre-registered, provide DOI in the manuscript**. For clinical trials, provide the trial registration number **OR** cite DOI.	Not Applicable	
Report the **clinical trial registration number** (at ClinicalTrials.gov or equivalent), where applicable.	Not Applicable	

Laboratory protocol	Information included in the manuscript?	In which section is the information available? (Reagents and Tools Table, Materials and Methods, Figures, Data Availability Section)
Provide DOI OR other citation details if **external detailed step-by-step protocols** are available.	Not Applicable	

Experimental study design and statistics	Information included in the manuscript?	In which section is the information available? (Reagents and Tools Table, Materials and Methods, Figures, Data Availability Section)
Include a statement about **sample size** estimate even if no statistical methods were used.	Yes	Figure 1C-F and Figure 5B
Were any steps taken to minimize the effects of subjective bias when allocating animals/samples to treatment (e.g. **randomization procedure**)? If yes, have they been described?	Not Applicable	
Include a statement about **blinding** even if no blinding was done.	Not Applicable	
Describe **inclusion/exclusion criteria** if samples or animals were excluded from the analysis. Were the criteria pre-established?<br><br>If sample or data points were omitted from analysis, report if this was due to attrition or intentional exclusion and provide justification.	Not Applicable	
For every figure, are **statistical tests** justified as appropriate? Do the data meet the assumptions of the tests (e.g., normal distribution)? Describe any methods used to assess it. Is there an estimate of variation within each group of data? Is the variance similar between the groups that are being statistically compared?	Yes	Figure legend of 5B

Sample definition and in-laboratory replication	Information included in the manuscript?	In which section is the information available? (Reagents and Tools Table, Materials and Methods, Figures, Data Availability Section)
In the figure legends: state number of times the experiment was **replicated** in laboratory.	Not Applicable	
In the figure legends: define whether data describe **technical or biological replicates**.	Yes	Figure 1C-F

Ethics

Ethics	Information included in the manuscript?	In which section is the information available? (Reagents and Tools Table, Materials and Methods, Figures, Data Availability Section)
Studies involving **human participants**: State details of **authority granting ethics approval** (IRB or equivalent committee(s), provide reference number for approval.	Not Applicable	
Studies involving **human participants**: Include a statement confirming that **informed consent** was obtained from all subjects and that the experiments conformed to the principles set out in the WMA Declaration of Helsinki and the Department of Health and Human Services Belmont Report.	Not Applicable	
Studies involving **human participants:** For publication of **patient photos**, include a statement confirming that consent to publish was obtained.	Not Applicable	
Studies involving experimental **animals**: State details of **authority granting ethics approval** (IRB or equivalent committee(s), provide reference number for approval. Include a statement of compliance with ethical regulations.	Not Applicable	
Studies involving **specimen and field samples:** State if relevant **permits** obtained, provide details of authority approving study; if none were required, explain why.	Not Applicable	

Dual Use Research of Concern (DURC)	Information included in the manuscript?	In which section is the information available? (Reagents and Tools Table, Materials and Methods, Figures, Data Availability Section)
Could your study fall under dual use research restrictions? Please check biosecurity documents and list of **select agents and toxins** (CDC): https://www.selectagents.gov/sat/list.htm	Not Applicable	
If you used a select agent, is the security level of the lab appropriate and reported in the manuscript?	Not Applicable	
If a study is subject to dual use research of concern regulations, is the name of the **authority granting approval and reference number** for the regulatory approval provided in the manuscript?	Not Applicable	

Reporting

The MDAR framework recommends adoption of discipline-specific guidelines, established and endorsed through community initiatives. Journals have their own policy about requiring specific guidelines and recommendations to complement MDAR.

Adherence to community standards	Information included in the manuscript?	In which section is the information available? (Reagents and Tools Table, Materials and Methods, Figures, Data Availability Section)
State if relevant guidelines or checklists (e.g., **ICMJE, MIBBI, ARRIVE, PRISMA**) have been followed or provided.	Not Applicable	
For **tumor marker prognostic studies,** we recommend that you follow the **REMARK** reporting guidelines (see link list at top right). See author guidelines, under 'Reporting Guidelines'. Please confirm you have followed these guidelines.	Not Applicable	
For **phase II and III randomized controlled trials**, please refer to the **CONSORT** flow diagram (see link list at top right) and submit the CONSORT checklist (see link list at top right) with your submission. See author guidelines, under 'Reporting Guidelines'. Please confirm you have submitted this list.	Not Applicable	

Data Availability

Data availability	Information included in the manuscript?	In which section is the information available? (Reagents and Tools Table, Materials and Methods, Figures, Data Availability Section)
Have **primary datasets** been deposited according to the journal's guidelines (see 'Data Deposition' section) and the respective accession numbers provided in the Data Availability Section?	Yes	Data and Code Availability Section
Were **human clinical and genomic datasets** deposited in a public access-controlled repository in accordance to ethical obligations to the patients and to the applicable consent agreement?	Not Applicable	
Are **computational models** that are central and integral to a study available without restrictions in a machine-readable form? Were the relevant accession numbers or links provided?	Yes	Data and Code Availability Section
If publicly available data were reused, provide the respective **data citations in the reference list**.	Yes	We have provided the accession numbers for public datasets, and the original papers have been appropriately cited in Main text and Reference.
